# Continuous intrathecal therapy for refractory cancer pain: A systematic review and meta-analysis focusing on partially implanted devices

**DOI:** 10.2340/1651-226X.2026.45778

**Published:** 2026-10-12

**Authors:** Mohammadhossein Radafshar, Maria E.C. Schelin, Elisabeth Bondesson, Sofia Löfvendahl, Eric Ahl, Sven Oredsson, Mikael Segerlantz

**Affiliations:** a Institute for Palliative Care, Department of Clinical Sciences Lund, Lund University and Region Skåne, Lund, Sweden; b Department of Palliative Care and Advanced Home Health Care, Region Skåne, Lund, Sweden; c Department of Clinical Sciences Lund, Orthopaedics, Lund University, Lund, Sweden; d HTA South, Department of Research, Development, Education and Innovation, Skåne University Hospital Lund, Lund, Sweden; e The Swedish Institute for Health Economics, Lund, Sweden

**Keywords:** Intrathecal, cancer pain, systematic review, meta-analysis, equipment and supplies

## Abstract

**Context:**

Conventional pain therapies sometimes fail in patients with severe neuropathic pain or bone metastases. Intrathecal therapy provides an alternative but is resource-intensive, technically demanding and costly.

**Objective:**

To compare the advantages and limitations of partially implanted devices (PIDs) versus conventional medical management or totally implanted devices (TIDs) in adults with cancer-related pain.

**Methods:**

A systematic literature review up to September 2024 was conducted, with an updated search performed in August 2026. Eligible studies included those on adults with refractory cancer pain receiving PID intrathecal therapy compared with conventional therapy, and comparative studies evaluating PIDs versus TIDs. Pain intensity was analysed using a meta-analysis; a synthesis without meta-analysis was used for other outcomes.

**Results:**

Of the 1,102 records, 15 were included, evaluating pain intensity, systemic opioid dose, quality of life (QoL), depression/anxiety, functional capacity and complications in PID-treated patients. A meta-analysis of pain intensity at 4 weeks showed statistically significant reduction with intrathecal therapy, which also reduced opioid use and improved QoL. Complication rates were lower than in conventional therapy. Investigations of depression/anxiety and functional capacity were limited, with inconsistent findings. Certainty of evidence was moderate for pain intensity and QoL; low for opioid dose, depression/anxiety and complications; and very low for functional capacity.

**Conclusions:**

Intrathecal therapy for refractory cancer pain with PIDs provides superior pain relief and improved QoL compared with the conventional therapy in patients with refractory cancer pain. Evidence for psychological and functional outcomes remains limited. Overall certainty of evidence is low to moderate, but findings support intrathecal therapy as a clinically meaningful option for selected patients.

**KEY MESSAGES**

**What is already known about the topic?**

- Intrathecal therapy is an effective treatment for refractory cancer pain and can reduce systemic opioid requirements.
- Evidence regarding partially implanted intrathecal devices has been reported in individual studies, but outcomes have not been synthesised across multiple clinically relevant domains.
- No previous systematic review has compared partially implanted and fully implanted intrathecal devices with respect to efficacy and complications.

**What this paper adds**

- No systematic review has comprehensively evaluated partially implanted intrathecal devices for the treatment of cancer pain across multiple clinically relevant outcomes, including quality of life, functional status, depression and anxiety, systemic opioid use and complications. In this review, we also compare partially implanted and fully implanted intrathecal devices with respect to pain intensity and complication rates.
- This systematic review and meta-analysis demonstrate that partially implanted intrathecal devices significantly reduce pain intensity and improve quality of life compared with conventional medical management.
- Partially implanted devices are associated with reduced systemic opioid use and lower complication rates than conventional therapy.
- No significant differences in pain relief or complications were found between partially and fully implanted intrathecal devices.

**Implications for practice, theory or policy**

- Partially implanted intrathecal devices represent a clinically effective option for selected patients with refractory cancer pain across diverse palliative care settings.
- Comparable outcomes between partially and fully implanted devices may support broader use of partially implanted systems, particularly in healthcare settings with limited resources or access to specialised interventions.
- These findings may inform clinical decision-making and service planning in palliative care internationally, where treatment choices must balance effectiveness, feasibility and resource availability.

## Introduction

Pain is one of the most prevalent, debilitating and feared symptoms amongst individuals living with cancer. It affects not only patients but also their families, often leading to significant emotional distress and a marked reduction in quality of life (QoL) [1]. Since the World Health Organisation (WHO) introduced the pain ladder for cancer pain management in 1986, significant progress has been made in alleviating suffering. However, numerous studies have shown that this stepwise approach is sometimes insufficient in severe cases and may be compromised by the adverse effects of analgesic therapy [2–4].

In approximately 10–15% of cases, cancer pain does not adequately respond to standard opioid and/or co-analgesic treatment. This refractory cancer pain is persistent, complex and difficult to manage, often requiring advanced and individualised strategies [4–6]. Treatment options may include opioid rotation or combination therapy, parenteral administration routes and the use of non-opioid agents such as nonsteroidal anti-inflammatory drugs (NSAIDs), antidepressants, anticonvulsants, N-methyl-D-aspartate (NMDA) receptor antagonists, lidocaine and corticosteroids [7]. However, the scientific evidence supporting the efficacy of these interventions remains limited [8].

Intrathecal drug delivery has long been recognised as an alternative approach for managing refractory cancer pain by delivering small amounts of medication directly into the cerebrospinal fluid, thereby bypassing the blood–brain barrier. Through a catheter placed in the spinal canal, one or more drugs are administered directly into the central nervous system via a pump. There are two techniques for pump placement: one involves a partially implanted device (PID) with the pump located outside the body, whilst the other involves a totally implanted device (TID) with the pump placed entirely within the body, without external connections. The choice between these techniques depends on factors such as available medical expertise, interdisciplinary collaboration, cost and the patient’s expected survival time.

### Complications and side effects

Complications and side effects vary significantly between conventional systemic treatment and intrathecal drug delivery. In traditional approaches, such as oral or intravenous opioids, patients often experience morphine-related side effects, including sedation, nausea, vomiting, constipation and risk of overdose. These adverse effects can impair QoL and may necessitate dose reduction or discontinuation, potentially compromising pain control [2, 4, 6]. The severity of these side effects is often a key reason for considering intrathecal therapy as a more targeted alternative [9].

Due to its different delivery route, intrathecal drug administration should allow for lower doses of opioids to achieve effective pain relief. As a result, many systemic side effects associated with high-dose opioids are reduced or minimised. However, this approach introduces its own set of risks, including infection at the implantation site, catheter-related complications, cerebrospinal fluid leaks and device malfunction. Neurological complications and drug-specific side effects may also occur [10–14].

### Evidence and rationale for this review

Effective management of cancer pain – especially in the palliative phase – is essential not only for symptom relief but also for preserving dignity, autonomy and psychosocial well-being. Addressing refractory pain often demands interdisciplinary collaboration, specialised expertise and a commitment to personalised care. Although intrathecal pain management has been used since the 1940s [15], a notable gap in the literature remains regarding the effectiveness and complications of intrathecal treatment for cancer pain, as well as the development and outcomes of various pump-placement techniques. A comprehensive comparative analysis of intrathecal treatment for cancer pain is therefore needed. To address this need and establish a scientific foundation for the potential benefits of intrathecal drug delivery within the Swedish healthcare context, a health technology assessment (HTA) analysis was conducted [16], forming the basis for this study.

The objective of this review is to evaluate existing research on the advantages and limitations of PIDs, versus either conventional medical management or TIDs, in adults with cancer-related pain. We aim to compare outcomes in terms of pain relief, opioid dosages, psychological well-being, QoL, activity levels, complications and side effects. This review is intended to generate insights that can inform clinical decision-making and enhance the management of cancer pain.

## Methods

This systematic review was conducted following the guidance of the Centre for Reviews and Dissemination (CRD) for healthcare reviews and was reported in accordance with the Preferred Reporting Items for Systematic Reviews and Meta-Analyses (PRISMA) 2020 statement.

### Search strategy

An initial search identified a recent HTA conducted by Health Ontario [17], which compared TIDs with conventional medical treatment for cancer pain. The methodology of this HTA served as the framework for our search strategy. A systematic literature search was conducted to identify clinical evidence, focusing on original articles addressing the use of PIDs for intrathecal drug delivery in cancer pain management. Searches were performed in MEDLINE (Ovid), Embase (Ovid) and CENTRAL (Cochrane Library via Wiley) in March 2024. To capture ongoing research, ClinicalTrials.gov and the WHO’s International Clinical Trials Registry Platform (ICTRP) were searched on 25 July 2024. All searches were updated on 20 September 2024, to include studies published during the project period.

An updated search was performed on 11 August 2026 using the original search strategy. This search yielded 197 additional records for screening. No additional studies met the inclusion criteria.

Automatic deduplication was performed in the Ovid search platform, followed by additional manual deduplication in Rayyan. Abstracts and full texts were screened independently by two reviewers (SO and EA), with any disagreements resolved through discussion or, when necessary, adjudication by a third reviewer (EB). Detailed search strategies and results are presented in Appendix A, and ongoing research is summarised in Appendix B.

A total of 15 articles focusing on PIDs were included for further analysis. Three of these articles were comparisons between TIDs and PIDs, in addition to comparison with conventional therapy (Appendix D). For these three articles, we analysed both the comparison between PIDs and conventional therapy (just the PID part was considered) and the comparison between PIDs and TIDs. All included studies were cross-checked against the Retraction Watch Database [18] to ensure that no retracted publications were analysed. A citation search using Web of Science was also conducted to identify additional relevant studies.

### Eligibility and exclusion criteria

Studies were selected based on the Population/Intervention/Control or Comparison/Outcome (PICO) criteria, as outlined in Table 1. The exclusion criteria are summarised in Table 2.

**Table 1:** PICO criteria.

PICO	Description
P	Adult patients with refractory cancer pain despite adequate conventional treatment
I	Intrathecal treatment with external pump
C	Conventional treatment
O	O1: Pain O2: Systemic opioid dose O3: Quality of life O4: Depression and anxiety symptoms O5: Functional performance O6: Complications and side effects

Note: P: population; I: intervention; C: control or comparison; O: outcome.

This PICO is used only for PID (Intrathecal treatment with external pump).

**Table 2:** Exclusion criteria.

Component	Limitation
Study design	Not a case report
Number of patients	≥ 30^a^
Type of publication	Not a conference abstract, conference review, letter, comment, editorial
Languages	English, Swedish, Danish, Norwegian
Year	Not a systematic review article published before 2015

a Rationale: This sample size restriction focused the review on studies with larger populations and greater statistical reliability.

### Risk of bias and certainty assessment

Two project leaders evaluated the risk of bias (RoB) in the included studies, and articles with an unacceptably high RoB were excluded. We used the National Institute of Health Quality Assessment Tool for Before-After (Pre-Post) Studies with no control group to evaluate RoB in the original articles. This appraisal tool was developed by the U.S. National Heart, Lung, and Blood Institute (NHLBI) for the quality assessment of pre-post studies without a control group [19].

In the comparison between external and internal pumps, three studies were identified as non-randomised comparative intervention studies (NRSI). For these studies, the RoB assessment was supplemented using the ROBINS-I tool [20], via the Swedish translation provided by the Swedish Agency for Health Technology Assessment and Assessment of Social Services (SBU) [21]. (Further details are provided in Appendix C.) Additionally, the Grading of Recommendations Assessment, Development and Evaluation (GRADE) system was applied to assess the certainty of evidence for each outcome, classifying evidence as high, moderate, low or very low, where ‘high certainty’ reflects robust confidence in the findings, ‘moderate’ indicates reasonable confidence, ‘low’ indicates limited evidence and ‘very low’ indicates no confidence.

### Data synthesis

For pain intensity, a meta-analysis was performed, since the data were sufficient and comparable. For other outcomes (i.e. opioid dose, QoL, psychological symptoms, functional performance and complications), a narrative synthesis or synthesis without meta-analysis (SWiM) methodology was applied to evaluate the reliability and certainty of evidence.

### Outcome 1: Pain intensity

Studies were assessed for clinical and methodological homogeneity in terms of population and study characteristics (Table 1). Eleven studies reported pain intensity before and after treatment, and nine of these used validated scales – the Visual Analog Scale (VAS) or the Numeric Rating Scale (NRS). Two studies [25, 31] employed alternative pain-assessment methods and were therefore excluded from the meta-analysis.

All the included studies but one [12], which focused solely on complications, enrolled patients with an inadequate response to conventional pain management. Seven studies additionally reported unacceptable side effects from systemic therapy as a reason for initiating intrathecal treatment.

The meta-analysis was conducted using the ‘metafor’ package in R Studio (v. 4.6-0). A random-effects model was applied to calculate the pooled mean difference in pain intensity during the first 4 weeks post-treatment compared with baseline. The between-study variance was estimated using the restricted maximum likelihood (REML) method.

Because several studies reported skewed data as medians with ranges or interquartile ranges, these values were converted to estimate means and corresponding measures of variability using established statistical methods. To facilitate interpretation, studies originally reporting medians and studies reporting means were also presented separately in subgroup analyses.

### Outcome 2: Systemic opioid use

Six studies (seven cohorts) reported systemic opioid doses before and after intrathecal treatment [11, 13, 14, 22–24]. In two studies [11, 13], systemic opioids were discontinued and substituted intrathecally following the initiation of therapy. In the remaining studies, systemic opioids were partially substituted intrathecally, and the systemic use partially continued. However, due to this heterogeneity in treatment strategies and reporting, a meta-analysis of systemic opioid use amongst the six included studies could not be performed.

### Outcome 3: Quality of life

Three studies assessed the effect of intrathecal therapy on health-related QoL using validated instruments: Euroqol’s five-dimension and -level questionnaire EQ-5D-5L [14], the Spitzer Quality of Life Index [13] and the European Organisation for Research and Treatment of Cancer (EORTC)’s QLQ-C30 questionnaire [10]. The use of different measurement scales precluded a meta-analysis, so the results of these three studies of QoL were synthesised narratively.

### Outcome 4: Depression and anxiety

Two studies reported symptoms of depression and anxiety but utilised different assessment tools [11, 14]: Ma et al. [11] used the Patient Health Questionnaire (PHQ-9) and Generalised Anxiety Disorder scale (GAD-7), whereas Giglio et al. [14] used the Edmonton Symptom Assessment System (ESAS). To facilitate comparison, scores were normalised to a 10-point scale, where 1 represents minimal symptoms and 10 represents severe symptoms (Appendix K, figure K3).

### Outcome 5: Functional capacity

Two studies assessed functional status before and after intrathecal therapy [25, 26]: Cheng et al. [25] employed a four-grade activity scale (bedridden, limited mobility, moderate mobility and free mobility), whereas Qin et al. [26] used the Karnofsky Performance Score (KPS). To enable cross-study comparison, both scales were converted to a 10-point scale (Appendix K, figure K4).

### Outcome 6: Complications and side effects

All studies, except for one by Fernández-Torres et al. [27], reported complications or adverse effects, albeit with variable detail. Complications were categorised as catheter-related or drug-related. A direct comparison of complications between intrathecal therapy and conventional treatment was not possible, as all the included studies employed a before-after design, and baseline side effects from conventional therapy were often inadequately reported.

## Results

### Study selection

After automatic and manual deduplication, 1,102 records remained for title and abstract screening. Following title and abstract screening, 55 records were deemed potentially relevant, and the full-text publications were retrieved. A full-text review identified 15 articles that met the inclusion criteria for this review. (Excluded studies are listed in Appendix E, an excluded review article is listed in Appendix F, an ongoing study is listed in Appendix G and the detailed results are listed in Appendix H.) The study-selection process is summarised in a PRISMA flow diagram (Figure 1).

**Figure 1:**
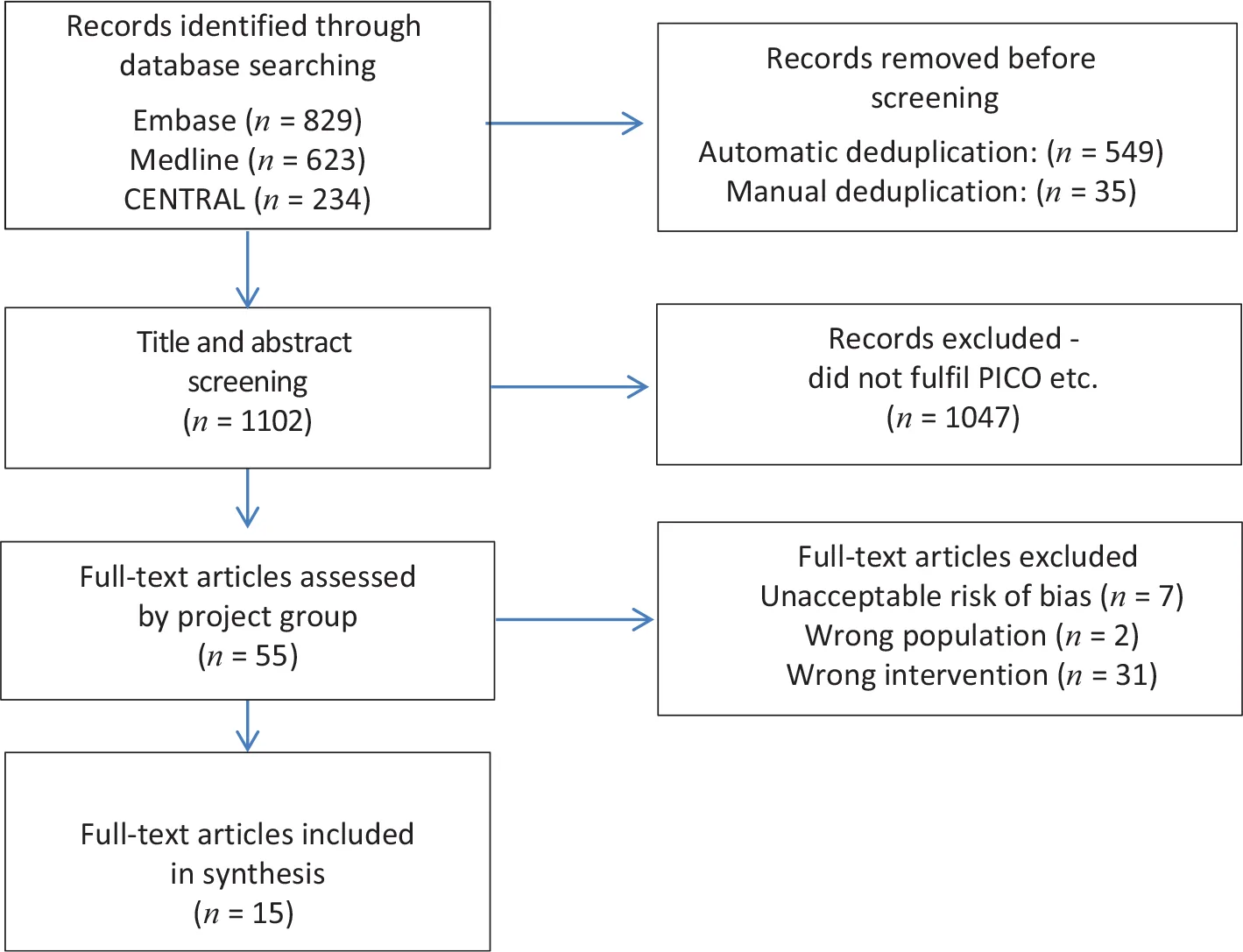
Flowchart according to PRISMA [28].

### Characteristics of the included studies

The 15 included articles reported on a total of 1,120 patients who received intrathecal drug delivery for cancer pain using a PID. Three of these articles were comparative, evaluating PIDs versus TIDs, whilst also functioning as before-after studies assessing the effectiveness of both device types relative to conventional therapy. For the main analysis, we used the data from the patients treated with PIDs to assess the six predefined outcomes.

From the comparative articles, including a total of 150 patients with TIDs and 205 with PIDs, we additionally evaluated the relative effectiveness and safety of PIDs versus TIDs, focusing on two specific outcomes: pain reduction and complications/side effects. Across the included studies, patients were typically eligible due to inadequate pain relief with conventional therapy or unacceptable side effects from systemic analgesics. The key characteristics of all included articles are summarised in Table 3.

**Table 3:** Characteristics of original articles (external pump).

Author, country	Study design/pain-assessment instrument	Multicentric (Mc) or monocentric (S)	Intervention	Comparison	Number of patients (gender); age	Cancer type	Follow-up time	Funding
Cheng et al., China [25]	Retrospective cohort/Expressive	S	IT via subcutaneous port (external pump)	NA	*n* = 100 (M = 41, F = 59); Mean age 49, (range 15–72)	Gastrointestinal, lung, breast, reproductive organs	12 weeks	Not specified
Díaz-Rodríguez et al., Spain [29]	Retrospective comparison/VAS	S	IT via subcutaneous port (external pump)	IT (internal pump)	*n* = 132; *I* = 60, *C* = 72 (M = 86, F = 46); Mean age 60.4 (SD ± 11.2)	Lung, colon, prostate, pancreas, urinary organs, breast	1, 3 and 6 months	No corporate funding
Ding et al., China [30]	Retrospective cohort/NRS	S	IT via subcutaneous port (external pump)	NA	*n* = 49 (M = 24, F = 25); Mean age 68.8 (SD ± 9.9)	Lung, colon, liver, pancreas, uterus, abdomen, urinary organs	At discharge, 1, 2, 3, 4, 5 and 6 months + 1 day before death	No corporate funding
Fernández-Torres et al., Spain [27]	Retrospective cohort^a^/VAS	S	IT via subcutaneous port (external pump) or internal pump	NA	*n* = 132 (external = 60, internal = 72) (M = 86, F = 46); Mean age 60.4 (SD ± 11.2)	Lung, colon, prostate, pancreas, urinary organs, breast	1, 3 and 6 months	No corporate funding
Giglio et al., Italy [14]	Prospective cohort/VASPI	S	IT via subcutaneous port (external pump)	NA	*n* = 50 (M = 34, F = 16); Mean age 69 (SD ± 12)	Pancreas, urinary organs, gastrointestinal, breast, liver, skin, lymphoma	2 weeks and 4 weeks	No corporate funding
Liu et al., China [10]	Retrospective cohort/VAS	S	IT via subcutaneous port (external pump)	NA	*n* = 84 (M = 38, F = 46); Mean age 55 (SD ± 13)	Lung, uterus, breast, liver, abdomen, colon	1, 2, 3, 6 and 9 months	No corporate funding
Ma et al., China [11]	RCT/VAS	Mc	IT (external pump) Hydromorphone	IT (external pump) Morphine	*n* = 233 (M = 152, F = 81); Mean age 60 (range 35–88)	NA (Proportion of skeletal metastases: 46.5% (I), 53.7% (C)	3 months	Funding from Science and Technology Commission in municipality
Madrid et al., Spain [31]	Retrospective cohort/Lattinen	S	IT via subcutaneous port (external pump)	NA	*n* = 35 (M = 24, F = 11); Mean age not specified (range 8–75)	Gastrointestinal, lung, breast, urinary organs, uterus, skeleton, mouth, lymphoma	1, 3 and 5 weeks	Not specified
Mercadante et al., Italy [22]	Prospective cohort/NRS	S	IT via subcutaneous port (external pump)	NA	*n* = 55 (M = 32, F = 23); Mean age 60 (range 33–82)	Urinary organs, lung, colon, breast	1, 3, 6 months + 1 week before death	Not specified
Neira et al., Spain [32]	Prospective comparison/VAS and Lattinen (focus on complications)	S	IT (external pump)	IT (Internal pump)	I: *n* = 42 (M = 24, F = 18); Mean age 59.8 (SD ±2.1); C: *n* = 42 (M = 32, F = 10); Mean age 57 (SD ±1.8)	Lung, uterus, prostate, abdomen	15–150 days	Not specified
Nitescu et al., Sweden [12]	Prospective cohort/Just looked at complications	S	IT (external pump)	NA	*n* = 200 (M = 93, F = 107); Median age 61 (range 18–91)	Not specified	All patients followed until death	No corporate funding
Qin et al., China [26]	Retrospective comparison/NRS	S	IT via subcutaneous port (external pump)	IT (Internal pump)	*n* = 139; I = 103, C = 36 (M = 78, F = 61); Mean age I = 62 C = 59	Gastrointestinal, lung, urinary organs, reproductive organs, liver	Unclear	No corporate funding
Sjöberg et al., Sweden [23]	Prospective cohort/NRS	S	IT (external pump)	NA	*n* = 52 (M = 21, F = 31); Median age 58 (range 21–81)	Pharynx, lung, abdomen, gallbladder, pancreas, colon, kidney, urinary organs, prostate, breast, uterus, reproductive organs, skin	6 months (multiple time points during)	Pumps from Pharmacia AB + funding from Meda AB
Sjöberg et al., Sweden [24]	Prospective cohort/NRS	S	IT (external pump)	NA	*n* = 53 (M = 27, F = 26); Mean age 62 (SD ±13)	Not specified	6 months (multiple time points during)	Not specified
Zheng et al., China [13]	Prospective cohort/NRS	S	IT via subcutaneous port (external pump)	NA	*n* = 53 (M = 29, F = 24); Mean age 66.4 (SD ± 18.5)	Lung, colon, liver, pancreas, prostate, pharynx, breast	3 months + 1 week before death	Not specified

RCT: Randomised clinical trial; VAS: Visual Analogue scale; NRS: Numeric Rating Scale; IT: Intrathecal treatment; I: intervention; C: comparison; F: female; M: male; SD: standard deviation; NA: not available.

a The same cohort as Diaz-Rodriguez et al. [29].

#### The study designs included

- Four comparative studies (TIDs vs. PIDs) [26, 27, 29, 32],
- Five retrospective cohort studies [10, 25, 27, 30, 31],
- Five prospective cohort studies [13, 14, 22–24] and
- One randomised controlled trial comparing intrathecal morphine versus hydromorphone [11].

### Outcomes of the included studies

#### Outcome 1: Pain Intensity

Ten studies (11 cohorts) reported pain intensity, with follow-up intervals ranging from 2 to 5 weeks; an interval of 4–5 weeks was the most common (Figure 2).

**Figure 2:**
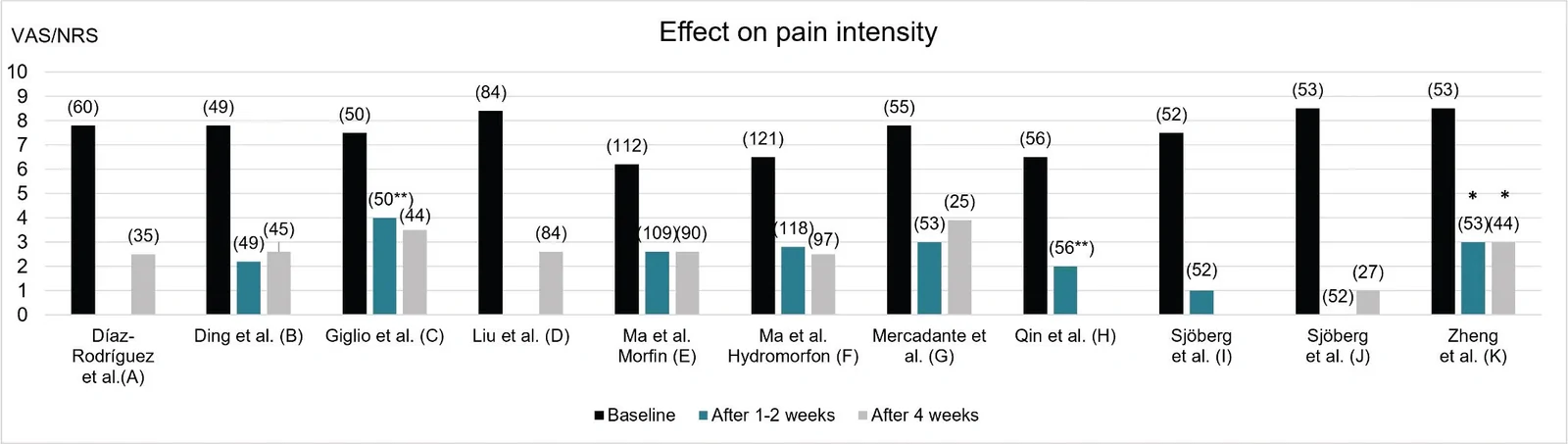
The effect of intrathecal treatment with external pump on pain, based on pain intensity scales reported in the original studies (VAS or NRS). Numbers in parentheses indicate study population*.* The assessment instrument used in each study is presented in Table 3 and Appendix H. *Significance (*P* < 0.05); **Study population is not mentioned and was assumed to be the same as baseline measurement. (A) Diaz-Rodriguez et al. [29]; (B) Ding et al. [30]; (C) Giglio et al. [14]; (D) Liu et al. [10]; (E&F) Ma et al. [11]; (G) Mercadante et al. [22]; (H) Qin et al. [26]; (I) Sjöberg et al. [23]; (J) Sjöberg et al. [24]; (K) Zheng et al. [13]. For clarity, only central estimates are displayed in the figures; measures of variability and uncertainty reported in the original studies are presented in Appendix H.

A meta-analysis of nine studies (10 cohorts) demonstrated a mean pain reduction of −4.8 points (95% confidence interval [CI]: −5.5 to −4.0) at 4 weeks post-implantation (Figure 3). Prediction intervals ranged from −7.2 to −2.4. A sensitivity analysis – excluding two studies that showed exceptionally large effects [13, 24] – resulted in a reduction of the pooled mean difference from −4.8 (95% CI: −5.5 to −4.0) to −4.3 (95% CI: −4.7 to −3.9). The overall effect therefore remained substantial, although the CI now approached the range considered a ‘large’ effect (Appendix I).

**Figure 3:**
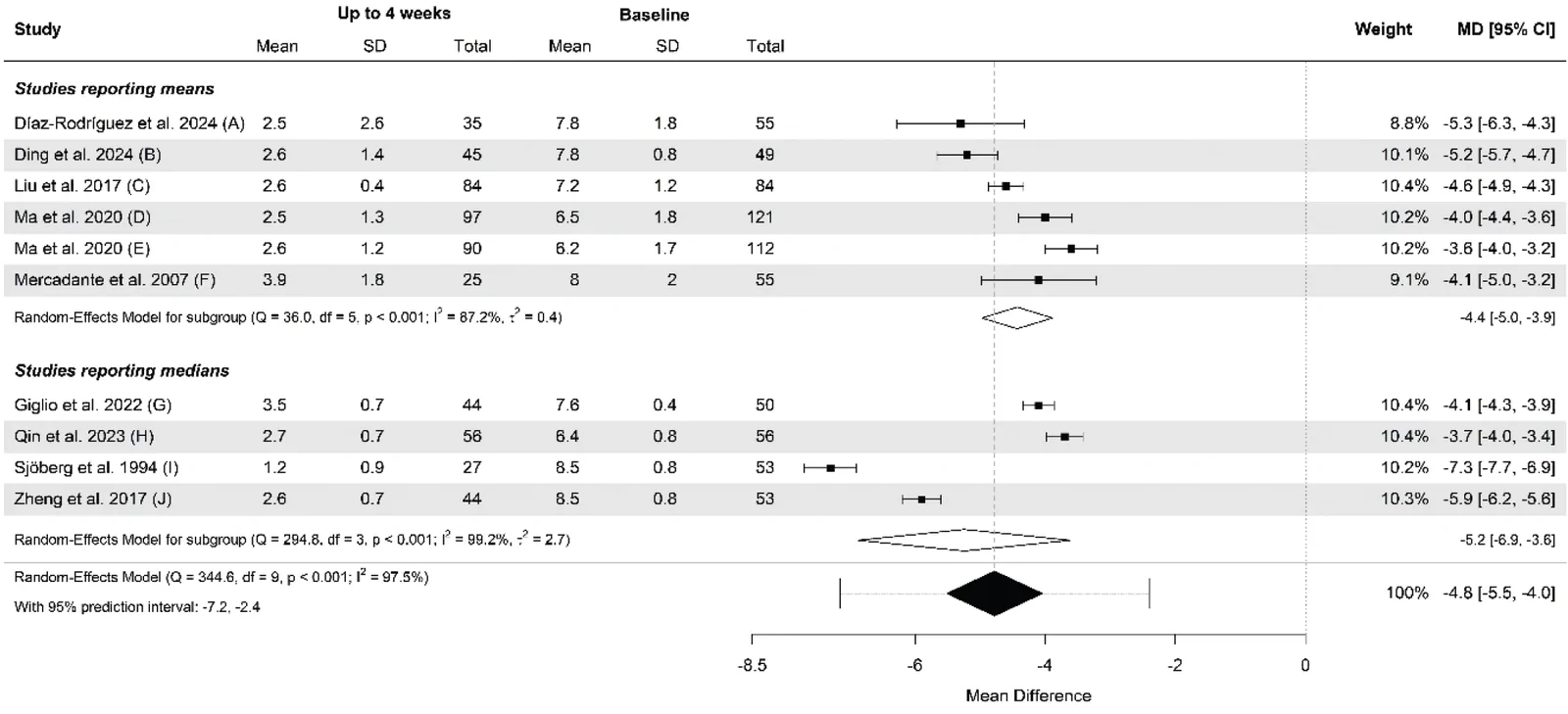
Difference in pain score measured up to 4 weeks after the implantation of intrathecal catheter with external pump. SD: standard deviation; MD: mean difference. (A) Diaz-Rodriguez et al. [29]; (B) Ding et al. [30]; (C) Liu et al. [10]; (D&E) Ma et al. [11]; (F) Mercadante et al. [22]; (G) Giglio et al. [14]; (H) Qin et al. [26]; (I) Sjöberg et al. [24]; (J) Zheng et al. [13].

According to the GRADE assessment, the results for pain intensity showed moderate certainty; it is probable that intrathecal therapy with an external pump significantly reduces pain compared with conventional treatment. The certainty was improved by the considerable effect size (Appendix J, table J1).

#### Outcome 2: Systemic opioid dose

Six studies (seven cohorts) reported systemic opioid use at baseline and follow-up (Appendix K, figure K1).

The narrative synthesis of the results on opioid usage in these studies indicated an average reduction of 279 mg/day in oral morphine equivalents. The GRADE assessment of the amount of systemic opioid doses before-after intrathecal treatment in these six studies indicated low certainty; thus, intrathecal therapy may reduce systemic opioid requirements, but heterogeneity and RoB limit confidence in this outcome (Appendix J, table J2).

#### Outcome 3: Health-related quality of life

Three studies evaluated QoL using different validated instruments: EQ-5D-5L, the Spitzer Quality of Life Index and EORTC QLQ-C30 (Appendix K, figure K2). All reported clinically meaningful improvements after 4 weeks. For example, improvements of 50% [10] and 125% [13] were observed. Positive effects persisted for up to 6–9 months, although the magnitude decreased over time.

A meta-analysis was not feasible due to the heterogeneous instruments, but a narrative synthesis indicated a substantial positive effect.

The GRADE assessment of the results of intrathecal pain management on QoL in these three studies showed moderate certainty; intrathecal therapy is likely to improve QoL compared with conventional treatment. The certainty was improved by the considerable effect size (Appendix J, table J3).

#### Outcome 4: Depression and anxiety

Two studies (three cohorts) assessed symptoms of depression and anxiety following intrathecal treatment (Appendix K, figure K3). Ma et al. [11] applied the PHQ-9 and GAD-7 scales, whereas Giglio et al. [14] used the ESAS for both depression and anxiety. Because of the small number of studies and the use of different measurement instruments, a meta-analysis was not feasible. Both studies reported reductions in symptoms, although the magnitude of improvement varied. The estimated reductions were 17–33% for depression and 0–25% for anxiety.

The certainty of evidence was rated as low, primarily due to the RoB and heterogeneity in the measurement instruments (Appendix J, table J4).

#### Outcome 5: Functional capacity

Two studies evaluated functional capacity before and after intrathecal treatment (Appendix K, figure K4). Cheng et al. [25] reported a meaningful improvement in mobility, with the proportion of patients classified as having *free activity* increasing from 8% at baseline to 28% after 1 week of treatment. In contrast, Qin et al. [26] observed no change in functional capacity.

Due to the small number of studies and inconsistent outcome measures (i.e. activity grade vs. KPS), a quantitative synthesis was not possible. The certainty of evidence was assessed as very low, downgraded for RoB, imprecision and inconsistency (Appendix J, table J5).

#### Outcome 6: Complications and side effects

All studies but one [27] reported complications and side effects, although the level of detail varied substantially. Complications associated with conventional treatment are common and well-known and often serve as an indication to consider intrathecal treatment as an alternative. However, these complications are reported inconsistently across studies, with varying levels of detail. Most complications are opioid-related. Given the substantial reduction in systemic opioid use following the initiation of intrathecal therapy, a corresponding decrease in these complications is expected.

Complications of intrathecal treatment can be categorised into two main groups:

1. *Catheter-related complications* were reported in a median of 3% of patients (range 0–14%) and included catheter slippage or occlusion, occasionally requiring removal. Infections (local, spinal or cerebral) occurred in a median of 1% of cases (range 0–12%), with isolated reports of meningitis [12, 22, 29, 31]. The most common minor complication following implantation was transient post-puncture headache.
2. *Drug-related side effects* were primarily due to intrathecal morphine and included constipation, nausea, vomiting and urinary retention. When local anaesthetics (e.g. bupivacaine) were added, neurological symptoms such as paraesthesia and gait disturbances were occasionally reported [12, 22–24].

As all the included studies employed a before-after design, direct comparison with conventional treatment was not feasible. No fatal adverse events were reported. The certainty of evidence was rated as low, primarily due to the observational study design and incomplete reporting (Appendix J, table J6).

### Comparison of intrathecal cancer pain management using TIDs versus PIDs

Three studies [26, 29, 32] directly compared the outcomes between patients receiving intrathecal therapy via TIDs versus those receiving it via PIDs (Tables 3 and appendix J table J7).

Díaz-Rodríguez et al. [29] conducted a retrospective comparative cohort study of 132 patients (60 PID vs. 72 TID). Group allocation was based partly on clinical judgement and expected survival. No significant differences were observed between the groups in pain reduction, nocturnal pain or breakthrough pain. The PID group experienced more total complications and more infection compared with the TID group (5% vs. 0%). Problems with the catheter were nearly double in the PID group (including needle displacement in the injection port), but the rates were equivalent after excluding needle displacement.

Qin et al. [26] retrospectively analysed outcomes in patients receiving a PID or TID, applying propensity score matching to correct for baseline imbalances. Post-treatment assessments (at hospital discharge, ~2 weeks) showed no significant differences in pain intensity, functional status or complication rates. A cost analysis indicated that, whilst initial placement of the PID was less expensive, the long-term maintenance costs were higher.

Neira et al. [32] compared 42 patients treated with PIDs and 42 with TIDs. Pain relief during the first 15 days was similar across groups. Side effects differed in nature rather than frequency, with a higher rate of infections and seromas in the PID group.

Across studies, the certainty of evidence was downgraded for bias and imprecision and rated as moderate for pain reduction and low for complications and side effects (Appendix J, tables 11 and 12, respectively).

### Conclusion regarding PIDs versus TIDs

It is possible that the overall risk of complications and side effects is comparable between TID and PID systems. Both methods provide similar pain relief, but their economic implications and maintenance requirements differ [9, 27].

## Discussion

The results of our meta-analysis regarding PIDs demonstrate a statistically and clinically significant effect of intrathecal treatment on refractory cancer pain, both within the time frame of up to 4 weeks after initiation and at the latest reported follow-up, compared with baseline. This finding is also reported in the three articles comparing PIDs and TIDs included in our review, as well as in the 2024 healthcare assessment performed in Ontario, Canada, regarding TIDs [17]. Although all included studies employed a before-and-after design, the certainty of evidence according to GRADE is rated as moderate because of the very strong effect size. This rating indicates that, although intrathecal treatment offers a strong reduction in pain intensity and a significant clinical effect, clinical decision-making should still proceed with appropriate caution whilst considering a multidisciplinary conference to ensure appropriate patient selection.

Nevertheless, the results should be interpreted with consideration of the heterogeneity amongst studies regarding patient populations, treatment protocols, outcome assessment and follow-up duration.

Similar patterns were observed for other outcomes. Improvements in health-related QoL (GRADE: moderate) and a reduced need for systemic opioids (GRADE: low) both showed clinically meaningful effects in the narrative synthesis. In contrast, the number of studies addressing functional capacity and anxiety/depression was limited, and the heterogeneity of assessment tools resulted in a very low certainty of evidence for these outcomes.

Furthermore, pain, fatigue, depression and sleep disturbances frequently form an interrelated symptom cluster in patients with advanced cancer, with each symptom potentially exacerbating the others. This complex interplay may influence both patients’ perception of pain and the assessment of health-related QoL. Consequently, the improvements observed in pain intensity following intrathecal treatment should be interpreted within the context of the broader symptom burden experienced by this patient population.

Our findings are largely consistent with previous research on intrathecal treatment for cancer pain using implanted pumps [17]. Both our analysis and such earlier studies demonstrate that intrathecal therapy is an effective option when conventional pain management is insufficient or when systemic side effects become intolerable.

Regarding the reduction or discontinuation of systemic opioids, the results are likely influenced by variations in opioid tapering protocols. In some studies, systemic opioids were fully converted to intrathecal administration and discontinued abruptly, whilst others implemented partial conversion, allowing the pain control from intrathecal therapy to guide further dose adjustments. Despite these methodological differences, which precluded a pooled meta-analysis for this outcome, the overall direction of the evidence suggests a consistent pattern: intrathecal treatment substantially reduces the need for systemic opioids.

Because the types of complications and side effects differ between conventional and intrathecal treatments, direct comparisons are difficult. In conventional therapy, morphine-related side effects are often severe – an issue that itself frequently motivates the transition to intrathecal administration. Whilst these systemic side effects tend to decrease with intrathecal treatment, other risks are introduced. Amongst the potential complications, infection remains the most serious, as it may necessitate discontinuation of treatment. However, with careful management and adherence to a strict aseptic technique, infection-related complications can be minimised. This recommendation may imply that, theoretically, some of the side effects associated with intrathecal treatment could be avoidable if appropriate precautions are taken, whereas the side effects of conventional treatment are largely unavoidable. Overall, the risk of complications and side effects associated with intrathecal therapy appears to be lower than that with conventional systemic treatment. Side effects caused by intrathecal medications can usually be managed by dose adjustment or switching to alternative agents, and many are transient and self-limiting. The risk of leg weakness increases if the catheter tip is positioned ventrally (in front of the spinal cord), highlighting the importance of using imaging verification for catheter placement [33].

The impact on anxiety and depression tends to be modest and decreases over time. Given that this patient group typically has a progressive and life-limiting disease, even small improvements in these outcomes may hold meaningful clinical relevance. Moreover, the fading effect on QoL with time that was observed in the studies is likely due to disease progression.

Unfortunately, intrathecal treatment of cancer pain, whether with PIDs or TIDs, remains relatively unknown amongst healthcare professionals working in palliative care; thus, many professionals are unlikely to recognise patients in need of such treatment, making appropriate patient selection a challenge. Successful implementation also requires close collaboration across different levels of care – from initial assessment and referral to a pain clinic, through the surgical implantation and stabilisation period in hospital, and finally to follow-up within advanced home care services. Regular maintenance, including weekly or biweekly dressing changes, needle replacement and refilling of the pump reservoir, is essential for safe and effective long-term management.

We investigated two outcomes (pain intensity and the occurrence of complications and side effects) in three studies identified in our review that compared PID with TID methods [26, 29, 32]. Regarding these two outcomes, the results were very similar (Tables 11 and 12).

An additional – but often overlooked – aspect of the comparison between PIDs and TIDs is the potential for increased independence and convenience provided by a fully implanted pump system that eliminates the need for external components. This feature may significantly enhance the QoL for both patients and their families, yet it is rarely investigated in the existing literature.

Looking ahead, there is a clear need for more precise guidelines to support the selection of patients suitable for intrathecal drug-delivery systems. Optimising treatment protocols, improving long-term outcomes and addressing logistical and ethical challenges in community and palliative care are essential next steps [9, 26].

The complexity of cancer-related pain and its impact on QoL is further illustrated by recent evidence reviews addressing neuropathic pain in cancer survivors, which also emphasise the need for individualised management strategies [34].

As double-blind randomised studies are not feasible due to ethical considerations in this context, multicentre prospective studies with standardised protocols would help generate evidence that is both reliable and applicable to clinical practice. Conducting a health economic evaluation would be highly valuable; however, its generalisability would potentially be limited, as healthcare costs and economic structures vary considerably across countries. Nevertheless, such an analysis – even if restricted to a single national context – could provide important insights. By presenting the methodology and cost components in detail, it would allow other countries to adapt and interpret the findings in accordance with their local economic conditions.

## Strengths and limitations of this study

All the studies reviewed herein employed a before-and-after design – a necessity, given the ethical and practical challenges of studying patients with progressive cancer disease. Whilst this design entails certain limitations, it should not overshadow the robust and clinically meaningful effects consistently demonstrated.

The existing literature is limited by patient selection bias, including overly optimistic outcome expectations, short survival times and insufficient evaluation of pre-implantation criteria and complications. A more structured analytical approach could help clarify the magnitude of treatment effects across different outcomes, identify the methodological shortcomings of existing research and guide the development of standardised clinical protocols for future studies.

The concept of refractory pain, which forms the primary indication for intrathecal therapy, remains poorly defined. Many studies also lack detailed documentation of the medications used before the initiation of intrathecal treatment, aside from opioid therapy. This may result in less comparable patient populations in the included articles.

A further limitation is the substantial loss to follow-up observed in several included studies. Because post-treatment outcomes were often available only for a subset of the original study population, attrition bias cannot be excluded. Patients with worsening clinical condition, insufficient pain relief, treatment discontinuation or death may have been less likely to contribute follow-up data, potentially leading to an overestimation of treatment effects.

Another limitation is that the meta-analysis was based on aggregate before-and-after data. As correlations between baseline and follow-up measurements were not consistently reported in the original studies, within-subject dependencies could not be accounted for in the analysis. This may have resulted in conservative estimates of uncertainty.

The RoB assessment of the included non-randomised studies revealed several concerns. All but one of these studies were single-centre investigations, and the only multicentre study was conducted within a single country, limiting the generalisability of the findings. Furthermore, substantial heterogeneity in data-collection methods and treatment techniques prevented a quantitative meta-analysis for several secondary outcomes.

## Conclusion

Once the decision is made to use intrathecal treatment for cancer pain, significantly improved pain control, a reduced need for systemic opioids and better health-related QoL can be expected. However, the effects of this treatment on anxiety, depression and functional capacity remain uncertain and require further investigation.

Although the available evidence supports intrathecal therapy as an effective option for refractory cancer pain, further interpretation of the findings should take into account the heterogeneity amongst studies and the methodological limitations of the current evidence base.

The interpretation of treatment outcomes should also acknowledge that cancer-related pain is often part of a broader symptom cluster involving fatigue, depression and sleep disturbances. Future studies should evaluate these interconnected symptoms alongside pain relief to provide a more comprehensive assessment of patient benefit and QoL.

Although the quality of evidence, as assessed by GRADE, is limited by the absence of randomised controlled trials, these limitations largely reflect the ethical and practical challenges inherent in this field rather than a lack of therapeutic benefit. When conventional treatments fail to provide adequate pain relief at the end of life, it would be neither medically nor ethically appropriate to withhold a potentially effective intervention such as intrathecal therapy.

## Supplementary Material



## Data Availability

This study is a systematic review and meta-analysis based on previously published data; no new patient data were collected. Data extracted from the included studies, the full search strategies and the risk-of-bias assessments are available in the Appendix accompanying this article. Additional materials, including analytic code, are available from the corresponding author upon reasonable request.
