# Supplementary material for: Continuous intrathecal therapy for refractory cancer pain: A systematic review and meta-analysis focusing on partially implanted devices
Source: Acta Oncol. 2026 Oct 12;65:45778. doi: 10.2340/1651-226X.2026.45778 (PMC13670362; doi:10.2340/1651-226X.2026.45778)
Supplement: Supplementary file 1 [file AO-65-45778-s1.pdf]

Supplementary material has been published as submitted. It has not been copyedited, or typeset by Acta Oncologica

Appendix A: Search strategies and database

Table A1: Search strategy for Embase via Ovid and MEDLINE via Ovid*

Embase 1974 to 2026 August 07

Ovid MEDLINE(R) ALL 1946 to August 10, 2026

Last search date: August 11th, 2026

#	Query
1	Cancer Pain/
2	((adenocarcinoma* or cancer* or carcinoma* or neoplasm* or oncolog* or tumor* or tumour* or malignan* or metasta*) adj10 (pain or pains or painful*)).ti,ab,kf.
3	or/1-2
4	Infusion Pumps, Implantable/
5	*Infusion Pumps/
6	*Injections, Spinal/
7	(intrathecal or intra-thecal).ti.
8	(((intrathecal* or intra-thecal* or implantabl*) adj3 (deliver* or device* or drug administrat* or infus* or pump* or system* or therap* or analgesi* or baclofen* or morphine* or ziconotide*)) or IDD or IDDS or ITDD or IADP).ti,ab,kf.
9	(target* drug deliver* or TDD).ti,ab,kf.
10	(subarachnoid adj4 (deliver* or drug* or infusion* or pump*)).ti,ab,kf.
11	(synchromed* or isomed* or infusaid* or constant flow infusion*).ti,ab,kf.
12	or/4-11
13	3 and 12
14	exp Animals/ not Humans/
15	13 not 14
16	15 use medall
17	Case Reports/ or Comment.pt. or Editorial.pt. or (Letter not (Letter and Randomized Controlled Trial)).pt. or Congress.pt.
18	16 not 17
19	limit 18 to (danish or english or norwegian or swedish)
20	cancer pain/
21	((adenocarcinoma* or cancer* or carcinoma* or neoplasm* or oncolog* or tumor* or tumour* or malignan* or metasta*) adj10 (pain or pains or painful*)).tw,kw,kf.
22	or/20-21
23	exp intrathecal pump/
24	implantable drug delivery system/
25	*drug delivery device/
26	*intraspinal drug administration/
27	(intrathecal or intra-thecal).ti,dv.
28	(((intrathecal* or intra-thecal* or implantabl*) adj3 (deliver* or device* or drug administrat* or infus* or pump* or system* or therap* or analgesi* or baclofen* or morphine* or ziconotide*)) or IDD or IDDS or ITDD or IADP).tw,kw,kf,dv.
29	(target* drug deliver* or TDD).tw,kw,kf,dv.

30	(subarachnoid adj4 (deliver* or drug* or infusion* or pump*)).tw,kw,kf,dv.
31	(synchromed* or isomed* or infusaid* or constant flow infusion*).tw,kw,kf,dv.
32	or/23-31
33	22 and 32
34	(exp animal/ or nonhuman/) not exp human/
35	33 not 34
36	35 use oemezd
37	Case Report/ or Comment/ or Editorial/ or (letter.pt. not (letter.pt. and randomized controlled trial/)) or conference abstract.pt. or conference review.pt.
38	36 not 37
39	limit 38 to (danish or english or norwegian or swedish)
40	19 or 39
41	remove duplicates from 40

*The search strategy was replicated from Ontario Health 2024. Modifications to the language filter (lines 19 and 39) as well as the database subsets (lines 16 and 36) were made.

Table A2: Search strategy for CENTRAL via Cochrane Library (Wiley)*

Last search date: August 11th, 2026

#	Query
1	MeSH descriptor: [Cancer Pain] this term only
2	((adenocarcinoma* OR cancer* OR carcinoma* OR neoplasm* OR oncolog* OR tumor* OR tumour* OR malignan* OR metasta*) NEAR/10 (pain OR pains OR painful*)):ti,ab,kw
3	#1 OR #2
4	MeSH descriptor: [Infusion Pumps, Implantable] this term only
5	MeSH descriptor: [Infusion Pumps] this term only
6	MeSH descriptor: [Injections, Spinal] this term only
7	(intrathecal OR intra-thecal):ti
8	(((intrathecal* OR intra-thecal* OR implantabl*) NEAR/3 (deliver* OR device* OR drug administrat* OR infus* OR pump* OR system* OR therap* OR analgesi* OR baclofen* OR morphine* OR ziconotide*)) OR IDD OR IDDS OR ITDD OR IADP):ti,ab,kw
9	(target* drug deliver* OR TDD):ti,ab,kw
10	(subarachnoid NEAR/4 (deliver* OR drug* OR infusion* OR pump*)):ti,ab,kw
11	(synchromed* OR isomed* OR infusaid* OR constant flow infusion*):ti,ab,kw
12	#4 OR #5 OR #6 OR #7 OR #8 OR #9 OR #10 OR #11
13	#3 AND #12

*The search strategy was replicated from Ontario Health 2024. The original search strategy was adapted to be performed in Cochrane Library via Wiley instead of Ovid.

Table A3. Searches for HTA documents and Cochrane Reviews

Citation searches in Web of Science Core Collection

Website/Organisation	Date	Query	Hits	Relevant
ClinicalTrials.gov	July 25, 2024	(cancer* OR mallignan*) AND intrathecal* AND pain*	52	1
ICTRP[a]	July 25, 2024	(cancer* OR mallignan*) AND intrathecal* AND pain*	43	1
ISRCTN Registry	July 25, 2024	(cancer* OR mallignan*) AND intrathecal* AND pain*	4	0
**Total**			**99**	**1 unique**

[a]ICTRP: International Clinical Trials Registry Platform

ditions: SCI-EXPANDED, SSCI, AHCI, CPCI-S, CPCI-SSH, BKCI,S, BKCI-SSH, ESCI

Search date: June 11, 2024
Seed references: [10-14,22-28,30]; Cheng et al.(25) was not indexed in Core Collection and was instead subject to reference list checking.

Table A4. Forward & backward citation and deduplication statistics

**Citation search type**	**Records**
Forward citation searching in Web of Science Core Collection	356
Backward citation searching in Web of Science Core Collection	717
**Total**	1073
Deduplicated records	504

APPENDIX B: Searches for ongoing clinical studies

Table B1. Search strategies for ongoing clinical studies.

Website/organisation	Date	Query	Hits	Relevant
ClinicalTrials.gov	July 25, 2024	(cancer* OR mallignan*) AND intrathecal* AND pain*	52	1
ICTRP[a]	July 25, 2024	(cancer* OR mallignan*) AND intrathecal* AND pain*	43	1
ISRCTN Registry	July 25, 2024	(cancer* OR mallignan*) AND intrathecal* AND pain*	4	0
**Total**			**99**	**1 unique**

[a]ICTRP: International Clinical Trials Registry Platform

Appendix C: Risk of bias

Table C1. Risk of bias assessed with the National Institutes of Health Quality Assessment Tool for Before-After (Pre-Post) Studies with No Control Group.

Criteria	Cheng et al.(25)	Ding et al.(30)	Díaz-Rodríguez et al.(29)	Fernández-Torres et al.(27)	Giglio et al.(14)	Liu et al.(10)	Ma et al.(11)	Madrid et al.(31)	Mercadante et al.(22)	Neira et al.(32)	Nitescu et al.(12)	Qin et al.(26)	Sjöberg et al.(23)	Sjöberg et al.(24)	Zheng et al.(13)
1. Was the study question or objective clearly stated?	No	Yes	Yes	Yes	Yes	Yes	Yes	Yes	Yes	CD	Yes	Yes	No	Yes	Yes
2. Were eligibility/selection criteria for the study population pre-specified and clearly described?	Yes	Yes	No	No	Yes	Yes	Yes	No	Yes	Yes	No	Yes	No	Yes	Yes
3. Were the participants in the study representative of those who would be eligible for the test/service/intervention in the general or clinical population of interest?	CD[a]	CD	CD	CD	CD	CD	CD	CD	Yes	CD	CD	CD	CD	CD	CD
4. Were all eligible participants that met the pre-specified entry criteria enrolled?	CD	CD	CD	CD	CD	CD	CD	CD	CD	CD	CD	CD	CD	CD	CD
5. Was the sample size sufficiently large to provide confidence in the findings?	CD	CD	CD	CD	CD	CD	Yes	No	CD	CD	CD	CD	CD	CD	CD
6. Was the test/service/intervention clearly described and delivered consistently across the study population?	No	Yes	Yes	CD	Yes	Yes	Yes	No	Yes	No	CD	Yes	No	CD	Yes
7. Were the outcome measures pre-specified, clearly defined, valid, reliable and assessed consistently across all study participants?	CD	Yes	Yes	Yes	Yes	Yes	Yes	No	Yes	Yes	CD	Yes	CD	Yes	Yes
8. Were the people assessing the outcomes blinded to the participants' exposures/interventions?	No	No	No	No	No	No	Yes	No	No	CD	No	No	No	No	No
9. Was the loss to follow-up after baseline 20% or less? Were those lost to follow-up accounted for in the analysis?	CD	Yes	NR	CD	Yes	Yes	Yes	CD	Yes	Yes	No	Yes	Yes	CD	Yes
10. Did the statistical methods examine changes in outcome measures from before to after the intervention? Were statistical tests done that provided *p* values for the pre-to-post changes?	CD	Yes	Yes	Yes	Yes	Yes	Yes	No	Yes	Yes	No	Yes	Yes	Yes	Yes
11. Were outcome measures of interest taken multiple times before the intervention and multiple times after the intervention (i.e. did they use an interrupted time-series design)?	No	No	No	No	No	No	CD	No	No	No	No	No	No	No	No
12. If the intervention was conducted at a group level (e.g. a whole hospital, a community, etc.), did the statistical analysis take into account the use of	NA[a]	NA	NA	NA	NA	NA	NA	NA	NA	NA	NA	NA	NA	NA	NA

individual-level data to determine effects at the group level?															
**Summary/conclusion (unacceptable poor, poor, fair, good)**	**Poor**	**Fair**	**Poor**	**Poor**	**Fair**	**Fair**	**Fair**	**Poor**	**Fair**	**Poor**	**Poor**	**Fair**	**Poor**	**Poor**	**Fair**

[a]CD = cannot determine, NA = not applicable

Table C2. Risk of bias assessed with Risk Of Bias In Non-randomized Studies of Interventions (ROBINS-I).

Domain	Díaz-Rodríguez et al.(29)	Neira et al.(32)	Qin et al.(26)
Bias due to confounding	Serious	Serious	Moderate
Bias in selection of participants into the study	Serious	Moderate	Moderate
Bias in classification of interventions	Serious	Serious	Moderate
Bias due to deviations from intended interventions	Low	Low	Low
Bias due to missing data	Serious	Moderate	Low
Bias in measurements of outcomes	Moderate	Moderate	Moderate
Bias in selection of reported results	Moderate	Low	Moderate
Conflict of interests[a]	No financial support	Not declared	Non-commercial financing
**Overall bias**	**Serious**	**Serious**	**Moderate**

[a]The assessment was made using a Swedish translation of the ROBINS-I tool by The Swedish Agency for Health Technology Assessment and Assessment of Social Services (SBU)(21). In this translation, a domain for risk of bias has been added.

Appendix D: Included articles – External pump

Table D1. Included articles

Included studies (original articles)	Risk of bias rating
**Cheng et al.(25)**<br>Cheng KI, Tang CS, Chu KS, Yip NS, Yu KL, Tseng CK. Retrospective investigation of intermittent bolus intrathecal morphine for cancer pain patients. Kaohsiung J Med Sci 1993;9(11):632-642.	Poor*
**Díaz-Rodríguez et al.(29)**<br>Diaz-Rodriguez D, Fontan-Atalaya IM, Peralta-Espinosa E, Fernandez-Torres B. Differences in efficacy and safety between intrathecal infusion devices in cancer pain. Pain Pract 2024;24(1):42-51. doi:10.1111/papr.13279	Poor*<br><br>Serious†
**Ding et al.(30)**<br>Ding Y, Deng H, Peng J. Clinical observation of the treatment of refractory cancer pain with cancer pain information platform and IDDS under home analgesia mode: a retrospective study. Medicine (Baltimore) 2024;103(27):e38765. doi:10.1097/md.0000000000038765	Fair*
**Fernández-Torres et al.(27)**<br>Fernandez-Torres B, Fontan-Atalaya IM, Peralta-Espinosa E, Diaz-Rodriguez D. Predictive factors for efficacy and safety of intrathecal infusion devices for oncological pain. Br J Pain 2023;17(6):569-578. doi:10.1177/20494637231198231	Poor*
**Giglio et al.(14)**<br>Giglio M, Preziosa A, Mele R, Brienza N, Grasso S, Puntillo F. Effects of an intrathecal drug delivery system connected to a subcutaneous port on pain, mood and quality of life in end stage cancer patients: an observational study. Cancer Control 2022;29:10732748221133752. doi:10.1177/10732748221133752	Fair*
**Liu et al.(10)**<br>Liu HJ, Li WY, Chen HF, Cheng ZQ, Jin Y. Long-term intrathecal analgesia with a wireless analgesia pump system in the home care of patients with advanced cancer. Am J Hosp Palliat Care 2017;34(2):148-153. doi:10.1177/1049909115615110	Fair*
**Ma et al.(11)**<br>Ma K, Jin Y, Wang L, et al. Intrathecal delivery of hydromorphone vs. morphine for refractory cancer pain: a multicenter, randomized, single-blind, controlled noninferiority trial. Pain 2020;161(11):2502-2510. doi:10.1097/j.pain.0000000000001957	Fair*
**Madrid et al.(31)**<br>Madrid JL, Fatela LV, Lobato RD, Gozalo A. Intrathecal therapy: rationale, technique, clinical results. Acta Anaesthesiol Scand Suppl 1987;85:60-7. doi:10.1111/j.1399-6576.1987.tb02671.x	Poor*
**Mercadante et al.(22)**<br>Mercadante S, Intravaia G, Villari P, et al. Intrathecal treatment in cancer patients unresponsive to multiple trials of systemic opioids. Clin J Pain 2007;23(9):793-798. doi:10.1097/AJP.0b013e3181565d17	Fair*
**Neira et al.(32)**	Poor*<br><br>Serious†

Neira F, Ortega J, Martinez F, Murillo H, CarrascoJimenez M. The Du Pen intrathecal catheter: alternative to multidose sequential mechanical infusion systems for management of cancer pain? Pain Clinic 1996;8(4):353-363.	
**Nitescu et al.(12)**<br>Nitescu P, Sjöberg M, Appelgren L, Curelaru I. Complications of intrathecal opioids and bupivacaine in the treatment of "refractory" cancer pain. Clin J Pain 1995;11(1):45-62. doi:10.1097/00002508-199503000-00006	Poor*
**Qin et al.(26)**<br>Qin W, Zhao L, Liu B, et al. Comparison of external system and implanted system in intrathecal therapy for refractory cancer pain in China: a retrospective study. Brain Behav 2023;13(1):e2851. doi:10.1002/brb3.2851	Fair*<br><br>Moderate†
**Sjöberg et al.(23)**<br>Sjoberg M, Appelgren L, Einarsson S et al. Long-term intrathecal morphine and bupivacaine in "refractory" cancer pain. Results from the first series of 52 patients. Acta Anaesthesiol Scand 1991;35(1):30-43. doi:10.1111/j.1399-6576.1991.tb03237.x	Poor*
**Sjöberg et al.(24)**<br>Sjöberg M, Nitescu P, Appelgren L, Curelaru I. Long-term intrathecal morphine and bupivacaine in patients with refractory cancer pain results from a morphine, bupivacaine dose regimen of 0.5:4.75 mg/ml. Anesthesiology 1994;80(2):284-297. doi:10.1097/00000542-199402000-00008	Poor*
**Zheng et al.(13)**<br>Zheng S, He L, Yang X, Li X, Yang Z. Evaluation of intrathecal drug delivery system for intractable pain in advanced malignancies: a prospective cohort study. Medicine (Baltimore) 2017;96(11):e6354. doi:10.1097/MD.0000000000006354	Fair*

*Assessed using the NHLBI Quality Assessment Tool for Before-After (Pre-Post) Studies with No Control Group (NHLBI)[19].

† Assessed with Risk Of Bias In Non-randomized Studies of Interventions (ROBINS-I)[20].

Appendix E: Excluded articles – External pump

Table E1. Excluded articles.

Excluded studies (original articles)	Motive for exclusion
Dupoiron D, Bienfait F, Carvajal G, Seegers V, Douillard T, Jubier-Hamon S, et al. Intrathecal cervical analgesia for cancer pain: a 12-year follow-up study in a comprehensive cancer center. Reg Anesth Pain Med 2023:rapm-2023-104961. doi: 10.1136/rapm-2023-104961	Wrong intervention
Ellis DJ, Dissanayake S, McGuire D, Charapata SG, Staats PS, Wallace MS, et al; Elan Study 95-002 Group. Continuous intrathecal infusion of ziconotide for treatment of chronic malignant and nonmalignant pain over 12 months: a prospective, open-label study. Neuromodulation 2008;11(1):40-49. doi: 10.1111/j.1525-1403.2007.00141.x	Wrong population
Erdine S, Yücel A. Long-term results of intrathecal morphine in 65 patients. Pain Clinic 1994;7(1):27-33.	Wrong intervention
Follett KA, Hitchon PW, Piper J, Kumar V, Clamon G, Jones MP. Response of intractable pain to continuous intrathecal morphine: a retrospective study. Pain 1992 Apr;49(1):21-25. doi: 10.1016/0304-3959(92)90183-C	Wrong population
Gestin Y, Vainio A, Pégurier AM. Long-term intrathecal infusion of morphine in the home care of patients with advanced cancer. Acta Anaesthesiol Scand 1997;41:12-17. doi: 10.1111/j.1399-6576.1997.tb04607.x	Unacceptable risk of bias
Kiehelä L, Hamunen K, Heiskanen T. Spinal analgesia for severe cancer pain: a retrospective analysis of 60 patients. Scand J Pain 2017;16:140-145. doi: 10.1016/j.sjpain.2017.04.073	Unacceptable risk of bias
Lazorthes Y, Verdie JC, Bastide R, Lavados A, Descouens D. Spinal versus intraventricular chronic opiate administration with implantable drug delivery devices for cancer pain. Appl Neurophysiol 1985;48(1-6):234-241. doi: 10.1159/000101133	Unacceptable risk of bias
Mitchell A, Somerville L, Williams N, McGhie J, McConnachie A, McGinn G, et al. More time in a community setting: a service evaluation of the impact of intrathecal drug delivery systems on place of care of patients with cancer pain. Palliat Med 2023;37(9):1461-1466. doi: 10.1177/02692163231191548	Wrong intervention
Mitchell A, Somerville L, Williams N, McGhie J, McConnachie A, McGinn G, et al. Implanted intrathecal drug delivery systems may be associated with improved survival in patients with cancer. Br J Pain 2024;18(2):110-119. doi: 10.1177/20494637231202089	Wrong intervention
Penn RD, Paice JA. Chronic intrathecal morphine for intractable pain. J Neurosurg 1987;67(2):182-186. doi: 10.3171/jns.1987.67.2.0182	Unacceptable risk of bias
Puntillo F, Giglio M, Preziosa A, Dalfino L, Bruno F, Brienza N, et al. Triple intrathecal combination therapy for end-stage cancer-related refractory pain: a prospective observational study with two-month follow-up. Pain Ther 2020;9(2):783-792. doi: 10.1007/s40122-020-00169-1	Wrong intervention
Schultheiss R, Schramm J, Neidhardt J. Dose changes in long- and medium-term intrathecal morphine therapy of cancer pain. Neurosurgery 1992;31(4):664-670. doi: 10.1227/00006123-199210000-00008	Wrong intervention

Staats PS, Yearwood T, Charapata SG, Presley RW, Wallace MS, Byas-Smith M, et al. Intrathecal ziconotide in the treatment of refractory pain in patients with cancer or AIDS: a randomized controlled trial. JAMA 2004;291(1):63-70. doi: 10.1001/jama.291.1.63	Wrong intervention
Van Dongen RTM, Crul BJP, De Bock M. Long-term intrathecal infusion of morphine and morphine/bupivacaine mixtures in the treatment of cancer pain: a retrospective analysis of 51 cases. Pain 1993;55(1):119-123. doi: 10.1016/0304-3959(93)90191-Q	Unacceptable risk of bias
Wang JK. Intrathecal morphine for intractable pain secondary to cancer of pelvic organs. Pain 1985;21(1):99-102. doi: 10.1016/0304-3959(85)90081-8	Unacceptable risk of bias

Appendix F: Excluded review article

Table F1. Excluded article.

Excluded studies (review articles)	Overall rating<br>Motive for exclusion
Perruchoud C, Dupoiron D, Papi B, Calabrese A, Brogan SE. Management of cancer-related pain with intrathecal drug delivery: a systematic review and meta-analysis of clinical studies. Neuromodulation 2023;26(6):1142-1152. Doi: 10.1016/j.neurom.2021.12.004	High risk of bias*

* As assessed by Ontario Health[17] using Risk of Bias in Systematic reviews (ROBIS) (Whiting 2016)(35)

Appendix G: Ongoing studies

Table G1. Ongoing studies.

Registration number	Study title	Locations of study	Estimated completion	Interventions	URL
NCT05674240	Prospective evaluation of intrathecal targeted drug delivery for cancer-associated pain	Oshkosh, Wisconsin, USA	2025-12-06	Medtronic SynchroMed™ II pump	https://clinicaltrials.gov/study/NCT05674240

Appendix H: Results

Table H1. Results: Effect on pain.

Author	Effect on pain
Cheng et al.[25]	VAS (0–100), proportion/level<br>*Baseline (n=100):*<br>VAS 81–100 (30%)<br>VAS 61–80 (52%)<br>VAS 41–60 (18%)<br>VAS 21–40 (0%)<br>VAS 10–20 (0%)<br>VAS 0–9 (0%)<br>*Up to 1 week:*<br>VAS 81–100 (0%)<br>VAS 61–80 (2%)<br>VAS 41–60 (3%)<br>VAS 21–40 (21%)<br>VAS 10–20 (54%)<br>VAS 0–9 (21%)<br>*Up to 4 weeks:*<br>VAS 81–100 (0%)<br>VAS 61–80 (2%)<br>VAS 41–60 (6%)<br>VAS 21–40 (23%)<br>VAS 10–20 (57%)<br>VAS 0–9 (12%)<br>*Latest (3 months):*<br>VAS 81–100 (0%)<br>VAS 61–80 (4%)<br>VAS 41–60 (7%)<br>VAS 21–40 (28%)<br>VAS 10–20 (52%)<br>VAS 0–9 (9%)
Díaz-Rodríguez et al.[29]	*VAS (0–10), mean (standard deviation)*<br>*Baseline (n=60): 7.82 (1.78)*<br>*Up to 1 week: data not available*<br>*Up to 4 weeks (n=35[a]): 2.50 (2.57[a]) (p<0.001)*<br>*Latest (3 months) (n=10[a]): 0.50 (0.85) (p<0.001)*<br>*[a]Supplementary data from Díaz-Rodríguez(36)*
Ding et al.(30)	*NRS (0–10), mean (± standard deviation[b])*<br>*Baseline (n=49): 7.84 (0.83)*<br>*At discharge (n=49): 2.16 (0.83)[c]*<br>*30 days after discharge (n=45): 2.58 (1.44)[cd]*<br>*60 days after discharge (n=36): 2.36 (1.37)[cd]*<br>*90 days after discharge (n=30): 2.33 (1.32)[cd]*<br>*120 days after discharge (n=21): 2.43 (0.68)[cd]*<br>*150 days after discharge (n=17): 2.29 (0.77)[cd]*<br>*180 days after discharge (n=15): 2.27 (0.70)[cd]*<br>*Day of patient's death (300–400 days) (n=49): 2.45 (0.61)[cd]*<br>*[b] Reported as standard deviation in the main text but as standard error of the mean in figures in the article. Treated by us as standard deviation.*<br>*[c] p<0.001 compared with baseline measurement*

	[d] *p>0.05 compared with time of discharge*
Giglio et al.[14]	*VAS (0–100), median (range)*<br>*Baseline (n=50): 75 (70–90)*<br>*Up to 1 week[e] (n=50[f]): 40 (30–60) (p<0.05)*<br>*Up to 4 weeks (n=44): 35 (20–50) (p<0.05)*<br>*Latest: see up to 4 weeks*<br>[e]*Refers to 2 weeks for this study*<br>[f]*Assumed to be the same sample size as at baseline*
Liu et al.[10]	*NRS (0–10), mean (standard deviation[g])*<br>*Baseline (n=84): 7.2 (1.2)*<br>*Up to 1 week: data not available*<br>*Up to 4 weeks (n=84): 2.6 (0.4) (p<0.05)*<br>*Latest (9 months) (n=21): 2.8 (0.4) (p<0.05)*<br>[g]*Incorrectly stated as standard error of the mean in the original publication*
Ma et al.[11]	*VAS (0–10), mean (standard deviation)*<br>*Morphine (ITMO)*<br>*Baseline (n=112): 6.2 (1.7)*<br>*Up to 1 week (n=109): 2.6 (1.2) (p<0.05)*<br>*Up to 4 weeks (n=90): 2.6 (1.2) (p<0.05)*<br>*Latest (3 months) (n=45): 2.4 (1.0) (p<0.05)*<br>*Hydromorphone (ITHM)*<br>*Baseline (n=121): 6.5 (1.8)*<br>*Up to 1 week (n=118): 2.8 (1.6) (p<0.05)*<br>*Up to 4 weeks (n=97): 2.5 (1.3) (p<0.05)*<br>*Latest (3 months) (n=48): 2.2 (1.2) (p<0.05)*
Madrid et al.[31]	*Number of patients with different intervals between injections, hours*<br>***During the 1st week (n=35):***<br>*24 hours: n=6*<br>*17–20 hours: n=19*<br>*12–14 hours: n=10*<br>*6 hours: n=0*<br>***During the 3rd week (n=35):***<br>*24 hours: n=2*<br>*17–20 hours: n=16*<br>*12–14 hours: n=10*<br>*6 hours: n=7*<br>***During the 5th week (n=31):***<br>*24 hours: n=0*<br>*17–20 hours: n=5*<br>*12–14 hours: n=12*<br>*6 hours: n=14*
Mercadante et al.[22]	*NRS (0–10), mean (95% CI)*<br>*Baseline (n=55): 7.98 (7.4–8.5)*<br>*Up to 1 week* (n=53): 3.0 (2.3–3.6) (p<0.0001)*<br>*Up to 4 weeks (n=25): 3.87 (3.1–4.6) (p<0.0001)*<br>*Latest (6 months) (n=3): 3.0 (0.5–5.5)*<br>**At discharge*
Qin et al.[26]	*NRS (0–10), median (interquartile range)*<br>*Baseline (n=56): 6.5 (6–7)*<br>*Up to 1 week (n=56[h]): 2 (2–3) (p<0.001)*<br>*Up to 4 weeks: data not available*<br>[h]*Sample size not specified, assumed to be the same as baseline*

Sjöberg et al.[23]	*VAS (0–10), number within each interval*<br>*Baseline (n=52):*<br>*0–2 (n=0)*<br>*3–6 (n=0)*<br>*7–8 (n=47)*<br>*9–10 (n=5)*<br>*Up to 1 week (n=52):*<br>*0–2 (n=44)*<br>*3–6 (n=8)*<br>*7–8 (n=0)*<br>*9–10 (n=0)*<br>*Up to 4 weeks: data not available*
Sjöberg et al. [24]	*VAS (0–10), median (interquartile range/range)*<br>*Baseline measurement (n=53): 8.5 (8–9/6–10)*<br>*Up to 1 week (n=52): 0 (0–1/0–2) (p<0.0001–0.007)*<br>*Up to 4 weeks (n=27): 1 (1–1/0–4) (p<0.0001–0.007)*<br>*Latest (6 months) (n=4): NA (0.5–1/NA)*
Zheng et al.(13)	*NRS (0–10), median (interquartile range/range)*<br>*Baseline (n=53): 8.5 (8–9/7–10)*<br>*Up to 1 week (n=53): 3 (2–3/1–4) (p<0.05)*<br>*Up to 4 weeks (n=44): 3 (2–3/1–4) (p<0.05)*<br>*Latest (3 months) (n=44): 3 (3–4/2–5) (p<0.05)*

Table H2. Results: Need for systemic opioids.

Author	Need for systemic opioids
Cheng et al.[25]	*Daily morphine dose, mean*<br>*Baseline[a] (n=100): 36.4 mg*<br>*Up to 1 week[b]: 0.32 mg*<br>*Up to 4 weeks[b]: 0.44 mg*<br>*Latest (3 months)[b]: 1.43 mg*<br>*[a]Oral*<br>*[b]Intrathecal (only)*
Giglio et al.[14]	*Daily morphine dose (morphine equivalents), mean (standard deviation)*<br>*Baseline (n=50): 360 (60) mg*<br>*Up to 1 week: data not available*<br>*Up to 4 weeks (n=44): 60 (standard deviation not specified) mg*
Ma et al.[11]	*Daily morphine dose OMEDD[c], median (range)*<br>*Morphine (ITMO):*<br>*Baseline (n=112): 201 mg (0–2088)*<br>*Hydromorphone (ITHM):*<br>*Baseline (n=121): 210 mg (0–2330)*<br>*Both cohorts:*<br>*Up to 1 week: 0[d]*<br>*Up to 4 weeks: 0[d]*<br>*[c]Oral morphine equivalent daily dose*<br>*[d]Patients were not allowed to take any pain-relieving treatment other than that given intrathecally.*
Mercadante et al.[22]	*Daily morphine dose (MOED[e]), mean (95% CI)*<br>*Baseline (n=55): 566 mg (435–697)*<br>*Up to 1 week (n=53): 25.7 mg (7.6–43.9)*<br>*Up to 4 weeks (n=25): 70 mg (17.8–122.1)*<br>*Latest (6 months) (n=3): 0 mg*<br>*[e]Morphine equivalent dose*
Qin et al.[26]	*Daily morphine dose OMED[f], median (range)*<br>*Baseline (n=103): 175 mg (80–600)*<br>*[f]Oral morphine equivalent dose*
Sjöberg et al.[23]	*Daily morphine dose, median (range)*<br>*Baseline (n=47): 110 mg (10–750)*<br>*Up to 1 week (n=45): 10 mg (0–500)*<br>*Up to 4 weeks (n=30): 15 mg (0–600)*<br>*Latest (6 months) (n=4): 50 mg*
Sjöberg et al. [24]	*Daily morphine dose, median (range)*<br>*Baseline (n=53): 120 mg (20–650)*<br>*Up to 1 week (n=52): 0 (0–500)*<br>*Up to 4 weeks (n=27): 10 (0–200)*<br>*Latest (6 months) (n=4): 10 (4–20)*
Zheng et al. (13)	*Daily morphine dose, median (range)*<br>*Baseline (n=53): 388 mg (219–1600)*<br>*Up to 1 week (n=53): 0*<br>*Up to 4 weeks (n=44): 0*<br>*Latest (3 months) (n=44): 10 mg*

Table H3. Results: Health-related quality of life.

**Author, year**	**Health-related quality of life**
Giglio et al.[14]	*EQ–5D–5L, median (range)*<br>*Baseline (n=53): -0.0089 (0.5160 to -0.1604)*<br>*Up to 1 week[a] (n=53): 0.5282 (-0.0076–0.07472)*<br>*Up to 4 weeks (n=44): 0.5418 (-0.0724–0.07472) (p<0.05)*<br>*[a]2 weeks*
Liu et al.[10]	*Spitzer Quality of Life Index (SQLI), mean (standard error of the mean)*<br>*Baseline (n=84): 5.5 (1.2)*<br>*Up to 1 week: data not available*<br>*Up to 4 weeks (n=84): 8.5 (1.2) (p<0.05)*<br>*Latest (9 months) (n=21): 5.5 (1.2)*
Zheng et al.(13)	*EORTC QLQ-C30: excellent (5), good (4), fair (3), poor (2), very poor (1), mean*<br>*Baseline (n=53): 1.5*<br>*Up to 1 week[a] (n=53): 3.9 (p<0.05)*<br>*Up to 4 weeks (n=44): 3.4 (p<0.05)*<br>*Latest (3 months) (n=44): 2.7 (p<0.05)*<br>*[a]At discharge*

Table H4. Results: Symptoms of anxiety and depression.

Author	Symptoms of anxiety and depression
Giglio et al.[14]	*Depression, (ESAS[a]), median (range)*<br><br>Baseline (n=50): 6 (4–8)<br>Up to 2 weeks[b] (n=50): 5 (3–7), ($p<0.05$)<br>Up to 4 weeks (n=44): 4 (3–6), ($p<0.05$)<br><br>*Anxiety, (ESAS), median (range)*<br><br>Baseline (n=50): 4 (2–7)<br>Up to 2 weeks[b] (n=50): 3 (2–6) ($p<0.05$)<br>Up to 4 weeks (n=44): 3 (2–6) ($p<0.05$)<br><br>[a]ESAS: Edmonton Symptom Assessment System<br>[b]First measurement after procedure
Ma et al.[11]	*Morphine (ITMO):*<br>*Baseline (n=112): 14.5*<br>*Up to 4 weeks (n=90): 12.5*<br>*Latest (3 months) (n=45): 10.5*<br>*Hydromorphone (ITHM):*<br>*Baseline (n=121): 14.5*<br>*Up to 4 weeks (n=97): 12.5*<br>*Latest (3 months) (n=48): 12*<br><br>*Anxiety (GAD-7 score) (0–30), mean[abc]*<br>*Morphine (ITMO):*<br>*Baseline (n=112): 9*<br>*Up to 4 weeks (n=90): 7.5*<br>*Latest (3 months) (n=45): 7*<br>*Hydromorphone (ITHM):*<br>*Baseline (n=121): 9*<br>*Up to 4 weeks (n=97): 8*<br>*Latest (3 months) (n=48): 9*<br><br>*[a]PHQ-9: Patient Health Questionnaire-9*<br>*[b]GAD-7: Generalized Anxiety Disorder-7*<br>*[c]It is not reported whether mean or median is used. Interpreted as mean.*

Table H5. Results: Functional capacity.

Author	Functional capacity
Cheng et al.[25]	*Activity impact, own scale*<br>*Baseline (n=100):*<br>*Bedridden (31%)*<br>*Limited activity (36%)*<br>*Moderate activity (25%)*<br>*Unrestricted activity (8%)*<br>*Up to 1 week (n=100*):*<br>*Bedridden (24%)*<br>*Limited activity (17%)*<br>*Moderate activity (31%)*<br>*Unrestricted activity (28%)*<br>**Assumed to be the same sample size as at baseline measurement*
Qin et al.[26]	*Functional ability, KPS score[a] (0–100), median (range)*<br>*Baseline (n=56): 92.5 (70–100)*<br>*Up to 1 week[b] (n=56): 90 (65–100)*<br>*[a]KPS: Karnofsky Performance Score*<br>*[b]At discharge*

Table H6. Results: Complications and side effects.

Author	Complications and side effects
Cheng et al.[25]	*Study population n=100*<br>*Early side effects (week 1):*<br>*Cauda equina syndrome (n=1)*<br>*Constipation (n=17)*<br>*Headache (n=18)*<br>*Itching (n=36)*<br>*Urinary retention (n=29)*<br>*Late side effects (week 2):*<br>*Anterior spinal artery syndrome (n=1)*<br>*Constipation (n=4)*<br>*Headache (n=2)*<br>*Catheter problems (n=8)*<br>*Meningitis (n=1)*<br>*Urinary retention (n=5)*
Díaz-Rodríguez et al.[29]	*Study population n=60*<br>*Infection (n=3)*<br>*Catheter migration (n=5)*<br>*Neurological impairment (n=3)*<br>*Needle dysfunction (n=7)*<br>*Spinal cord compression (n=1)*<br>*Other complications/side effects (n=6)*
Ding et al.(30)	*Study population n=49*<br>*Baseline (n=49):*<br>*Constipation[a]: 34*<br>*Nausea and vomiting[b]: 47*<br>*At discharge (n=49):*<br>*Constipation[a]: 16*<br>*Nausea and vomiting[b]: 4*<br>*30 days after discharge (n=43):*<br>*Constipation[a]: 9*<br>*Nausea and vomiting[b]: 6*<br>*60 days after discharge (n=36):*<br>*Constipation[a]: 10*<br>*Nausea and vomiting[b]: 6*<br>*90 days after discharge (n=30):*<br>*Constipation[a]: 7*<br>*Nausea and vomiting[b]: 7*<br>*120 days after discharge (n=21):*<br>*Constipation[a]: 11*<br>*Nausea and vomiting[b]: 7*<br>*150 days after discharge (n=17):*<br>*Constipation[a]: 6*<br>*Nausea and vomiting[b]: 2*<br>*180 days after discharge (n=15):*<br>*Constipation[a]: 7*<br>*Nausea and vomiting[b]: 2*<br>*Day of patient's death (300–400 days) (n=49):*<br>*Constipation[a]: 0*<br>*Nausea and vomiting[b]: 49*<br>*[a]Requires medication*<br>*[b]Unable to eat normally*<br>*Postoperative complications:*<br>*Cerebrospinal fluid leakage: 1*<br>*Wound infection: 3*
Giglio et al.[14]	*Study population n=50*<br>*Confusion (n=2)*<br>*Nausea (n=3)*

	Vomiting (n=2)<br>*Early urinary retention (n=4)*
Liu et al.[10]	*Study population n=84*<br>*Early side effects:*<br>*Constipation (n=22)*<br>*Nausea (n=56)*<br>*Itching (n=3)*<br>*Vomiting (n=14)*<br>*Difficulty urinating (n=29)*<br>*Fatigue (n=5)*<br>*Late side effects (month 1–2):*<br>*Constipation (n=19)*<br>*Nausea (n=6)*<br>*Itching (n=2)*<br>*Vomiting (n=2)*<br>*Difficulty urinating (n=2)*<br>*Fatigue (n=3)*
Ma et al.[11]	*Study population n=233*<br>*Respiratory effects (n=5)*<br>*Anorexia (n=40)*<br>*CSF leakage (n=2)*<br>*Constipation (n=62)*<br>*Nausea (n=75)*<br>*Infection or other problems from port pocket (n=5)*<br>*Catheter problems (n=4)*<br>*Itching (n=6)*<br>*Vomiting (n=53)*<br>*Reduced sensation (n=3)*<br>*Pump problems (n=8)*<br>*Urinary retention (n=45)*<br>*Dizziness (n=34)*
Madrid et al.[31]	*Study population n=35*<br>*Early side effects:*<br>*Respiratory effects (n=1)*<br>*Itching (n=7)*<br>*Vomiting (n=2)*<br>*Local bleeding (n=4)*<br>*Neck pain (n=5)*<br>*Urinary retention (n=11)*<br>*Late side effects:*<br>*Fistula (cerebrospinal fluid) (n=3)*<br>*Catheter occlusion (n=1)*<br>*Meningitis (n=1)*
Mercadante et al.[22]	*Study population n=55*<br>*Early complications[c]:*<br>*Headache (n=4)*<br>*Minor bleeding (n=2)*<br>*Reoperation (n=1)*<br>*Urinary retention (n=6)*<br>*Change of catheter entry site (n=3)*<br>*Late complications[c]:*<br>*Local infection (n=1)*<br>*Catheter infection (n=1)*<br>*Spinal compression (n=1)*<br>[c]*Related to catheter insertion*
Neira et al.[32]	*Study population n=42*<br>*CSF fistula (n=1)*<br>*Fever (n=1)*<br>*Headache (n=1)*<br>*Infection around reservoir (n=1)*<br>*Seizures (n=1)*<br>*Seroma (n=5)*<br>*Wound infection (n=4)*

	Pressure ulcer (n=1)<br>*Changed catheter position (n=6)*
Nitescu et al.[12]	*Study population n=200*<br>*Bleeding in catheter tunnel (n=1)*<br>*CSF accumulation (hygroma) (n=3)*<br>*Epidural hematoma (n=3)*<br>*Skin necrosis at puncture site (n=4)*<br>*Infection at puncture site (n=1)*<br>*Catheter obstruction (n=2)*<br>*Leakage from external catheter (n=7)*<br>*Infusion fluid leakage (n=3)*<br>*Meningitis (n=1)*<br>*Nerve root injury (n=2)*<br>*Unintentional removal of catheter (n=8)*<br>*Insufficient spread of medication (n=1)*<br>*Puncture headache (n=31)*<br>*Pressure ulcer (n=2)*<br>*Changed catheter position (n=3)*
Qin et al.[26]	*Study population n=56*<br>*Withdrawal symptoms (n=1)*<br>*Diarrhea (n=4)*<br>*Nausea and vomiting (n=18)*<br>*Post-surgical infection (n=2)*<br>*Post-puncture headache (n=1)*<br>*Urinary retention (n=9)*<br>*Dizziness (n=2)*<br>*Overdose symptoms (n=1)*
Sjöberg et al.[23]	*Study population n=52*<br>*Cerebral clonus (n=1)*<br>*Hypotension (n=5)*<br>*Complete paraparesis (n=1)*<br>*Motor disturbances, e.g., gait disturbance (n=3)*<br>*Paresthesia (n=2[d])*<br>*Urinary retention (n=9)*<br>*[d]Only patients who received bupivacaine (n=33)*
Sjöberg et al.[24]	*Study population n=53*<br>*Hypotension (n=1)*<br>*Motor impairment, e.g., gait disturbance (n=9)*<br>*Paresthesia (n=11[e])*<br>*Early urinary retention (n=14)*<br>*Late urinary retention (n=7)*<br>*Complications related to the surgical part are referred to another article(37, 38)*<br>*[e]Only patients who received bupivacaine (n=27)*
Zheng et al.(13)	*Study population n=53*<br>*Mild weakness in lower extremities (n=3)*<br>*Urinary difficulties (n=5), of which one patient had urinary retention*

Appendix I: Sensitivity analysis

A sensitivity analysis (see figure I1) of the outcome measure ‘effect on pain’ was conducted to examine how the results were influenced by two studies that showed a comparatively very large effect(13, 24). The sensitivity analysis shows a decrease in the overall mean from VAS –4.8 (CI: –5.5, –4.0) to –4.3 (CI: –4.7, –3.9). According to the project group's assessment, a change in VAS or NRS of 2–2.9 is considered ‘moderate’, 3–3.9 is ‘large’, and more than 4 scale points is ‘very large’, which means that the mean value still falls within the ‘very large’ category. However, it should be noted that the CI now borders on the range for a ‘large effect’. In summary, the project group’s assessment is that the sensitivity analysis indicates that the effect remains substantial even when the two studies showing the greatest effect are excluded.

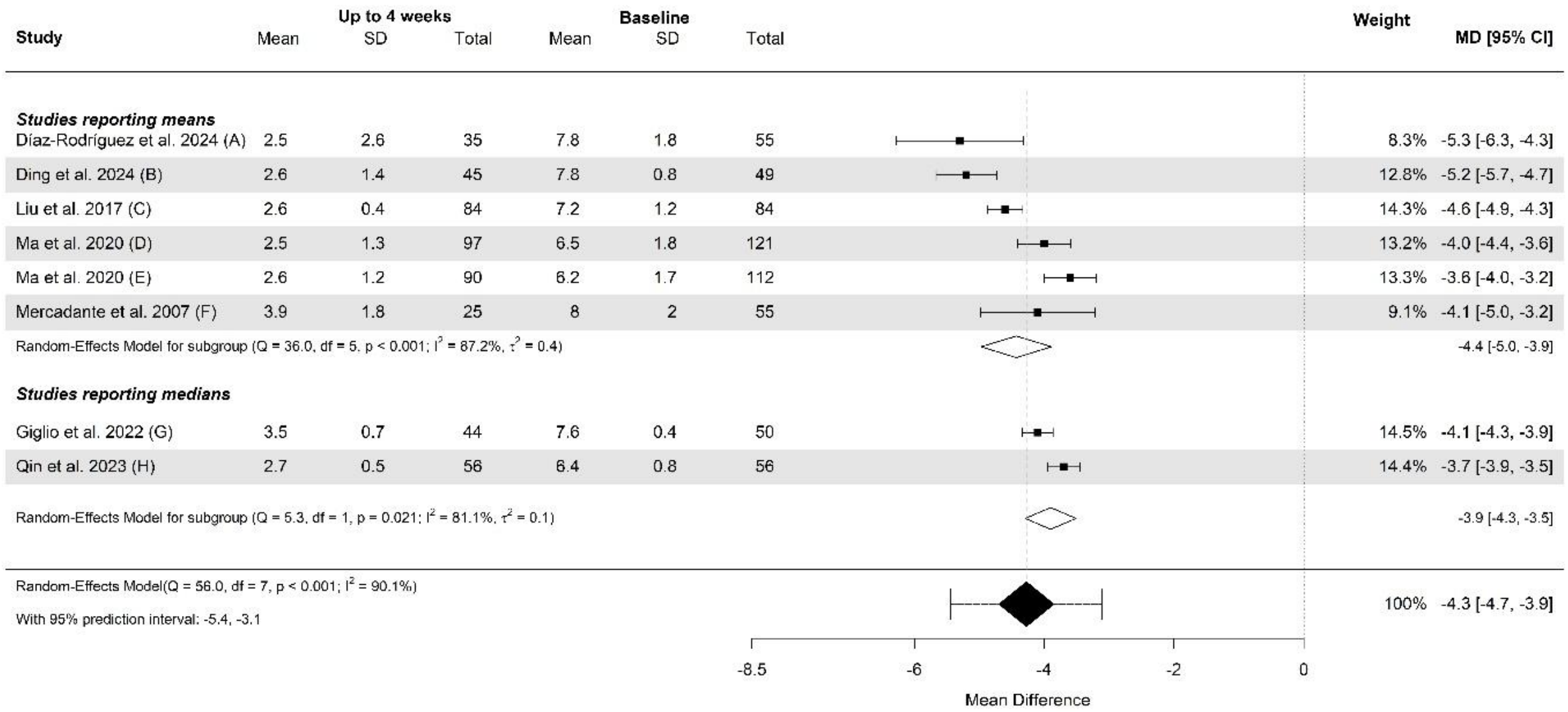

Figure I1: Sensitivity analysis when Sjöberg et al.(24) and Zheng et al.(13) are excluded from the meta-analysis.

Appendix J: Tables

Table J1. GRADE assessment of evidence reliability from 10 studies(10, 11, 13, 14, 22-24, 26, 29, 30) on changes in pain intensity following intrathecal treatment with external pump.

Outcome	Number of patients and studies	Results weighed together	reliability	Comment

**Intrathecal treatment of pain with an external pump compared with conventional treatment (before/after)**				
**Change by 10-grade scale (VAS/NRS)**	*n*=547, 9 observational studies (10 cohorts)	Intrathecal treatment with external pump diminishes the pain by an average of 4.8 points	⊕⊕⊕◯ Moderate reliability with a very strong effect on pain	Reliability assessment starts on +2 because of moderate-to-high risk of bias<br><br>Effect size: +1
**Conclusion: It is probable that intrathecal treatment of cancer pain with an external pump reduces the pain compared with conventional treatment, and the effect is very strong.**				

Table J2. GRADE assessment of evidence reliability for the effect of intrathecal treatment with an external pump in six studies(11, 13, 14, 22-24) on systemic opioid use.

Outcome	Number of patients and studies	Results weighed together	Reliability	Comment
**Intrathecal treatment of pain with an external pump compared with conventional treatment (before/after)**				
**Systemic opioid dose (mg/d)**	*n*=490, 6 observational studies	Intrathecal treatment with external pump diminishes the systemic opioid dose on average by 279 mg/day	⊕⊕◯◯ Low reliability, with a strong effect on systemic opioid dose	Reliability assessment starts on +2 because of moderate-to-high risk of bias

**Conclusion: It is possible that intrathecal treatment of cancer pain with an external pump reduces the systemic dose of opioids compared with conventional treatment, and the effect is strong.**

Table J3. GRADE assessment of the reliability of evidence from three studies(10, 13, 14) evaluating intrathecal external-pump treatment and health-related QoL.

Outcome	Number of patients and studies	Results weighed together	Reliability	Comment
**Intrathecal treatment of pain with an external pump compared with conventional treatment (before/after)**				
**Change in health-related QoL based on EQ-5D-5L, Spitzer QoL score or QLQ-C30 score**	*n*=290, 3 observational studies	Intrathecal treatment with an external pump increases health-related QoL	⊕⊕⊕◯ Moderate reliability, with a very strong effect on health-related QoL	Reliability assessment starts on +2 because of moderate-to-high risk of bias<br><br>Effect size: +1
**Conclusion: It is probable that intrathecal treatment of cancer pain with an external pump increases health-related QoL compared with conventional treatment, and the effect is very strong.**				

Table J4. GRADE assessment of evidence reliability for the effect of intrathecal treatment with an external pump on depression and anxiety in two studies.(11, 14)

Outcome	Number of patients and studies	Results weighed together	Reliability	Comment
**Intrathecal treatment with external pump compared** with **conventional treatment (before-after)**				

**Change in symptoms of depression and anxiety**	*n*=283, 2 observational studies (3 cohorts)	Intrathecal treatment with an external pump reduces depression symptoms by 17%–33% and anxiety symptoms by 0%–25%.	⊕⊕◯◯ Low reliability for a minor effect on depression and anxiety symptoms	The reliability assessment starts at +2 based on moderate or high risk of bias
**Conclusion: It is possible that intrathecal treatment with an external pump reduces symptoms of depression and anxiety compared with conventional treatment, but the effect is small.**				

Table J5. GRADE assessment of evidence reliability from two studies(25, 26) on the effect of intrathecal treatment with an external pump on functional capacity.

Outcome	Number of patients and studies	Results weighed together	Reliability	Comment
**Intrathecal treatment with external pump compared with conventional treatment (before-after)**				
**Change in functional ability**	*n*=156, 2 observational studies	It cannot be demonstrated that intrathecal treatment with an external pump affects functional ability.	⊕◯◯◯ Very low reliability that intrathecal treatment affects functional ability	The reliability assessment starts at +2 based on moderate or high risk of bias; Lack of precision: –1
**Conclusion: No conclusion can be drawn about whether intrathecal treatment with an external pump affects functional ability compared with conventional treatment.**				

Table J6. GRADE assessment of evidence reliability from 14 studies(10-14, 22-26, 29-32) on complications and side effects associated with intrathecal external-pump treatment

Outcome	Number of patients and studies	Results weighed together	Reliability	Comment
**Intrathecal treatment with external pump** compared with **conventional treatment (before-after)**				
**Risk of complications and side effects**	*n*=1073, 13 observational studies	Intrathecal treatment with an external pump has fewer side effects and complications.	⊕⊕◯◯ Low reliability that intrathecal treatment has fewer complications and side effects.	The reliability assessment starts at +2 based on moderate or high risk of bias.
**Conclusion: It is possible that intrathecal treatment with an external pump, compared** with **conventional treatment, has fewer side effects and complications, but the effect size is uncertain.**				

Table J7. Included studies comparing external and internal pumps.

Author and country	Study type, randomisation inclusion	Cohort 1 (intervention)	Cohort 2 (control)	Follow-up interval, outcome measures	Result	Comments
**Díaz-Rodríguez et al., Spain(29)**	Comparative retrospective cohort study *n*=136 Allocation between cohorts was determined by the treating physician.	PID (usually patients with an expected survival of <3 months) *n*=60	TID (usually patients with an expected survival of >3 months) *n*=72	Before-after at 1, 3 and 6 months. VAS (0–10) KPS (0–10), only before	Effect on pain: No significant difference between groups (*p*=0.074) VAS (mean difference) after 1 month: external, 5.25 scale points; internal, 4.43 scale points	Patients from 2009 to 2017 included. Intrathecal morphine dose was similar between groups. The need for a systemic

	Characteristics were similar between groups. KPS was higher in the internal cohort than in the external cohort.				No significant difference in nocturnal pain or episodic pain. Side effects and complications: external, 43.3%; internal, 25.0% (*p*=0.026) Excluding needle dysfunction: external, 28.3%; internal, 25.0%	opioid dose was also similar. Internal pumps more often required hospital care for complications (*p*=0.038). Mortality after 6 months: external, 82.5%; internal: 66% Infections, neurological complications and spinal cord compression were very rare and similar between groups.
**Neira et al., Spain(32)**	Comparative prospective cohort study *n*=84 Method of allocation between groups not described.	PID<br><br>*n*=42	TID<br><br>*n*=42	Before-after at 24, 48 and 72 hours, then every other day until day 15 Side effects and complications monitored for 50 days VAS (reported only at baseline)	Effect on pain – No significant difference in 'insufficient pain relief': external, 23.8%; internal, 14% (NS) Side effects and complications – wound infection: external, 9.5%; internal, 0% (*p*<0.05) Seroma: external, 9.5%; Internal, 0% (*p*<0.05) For all other side effects/complications, no significant difference between groups.	Patients from before 1995 included
**Qin et al.,**	Comparative retrospectiv	PID<br><br>*n*=103	TID<br><br>*n*=36	Baseline (at admission) and follow-	Effect on pain: No significant	Patients from 2019–

**China(26)**	e cohort study<br>Data from medical record review<br>*n*=139<br>After propensity score matching,<br>*n*=89	After propensity score matching,<br>*n*=56	After propensity score matching,<br>*n*=33	up (at discharge)<br>Implementation of external pump was faster (13 vs. 19 days, *p*<0.01)<br>VAS (0–10)<br>KPS (0–10)	difference between groups<br>VAS (mean reduction) at discharge: external, 4.5 scale points; internal, 5 scale points<br>Side effects and complications: No significant difference between groups<br>Costs: External pump has lower initial implementation cost (*p*<0.001) but higher monthly maintenance cost (*p*<0.001)	2021 included<br>High functional status in both groups (KPS=90)<br>Lower incidence of infections in patients with external pumps compared with previous studies, partly due to increased use of subcutaneous ports.

Table J8. GRADE assessment of evidence reliability from three studies(26, 29, 32) on pain outcomes comparing PIDs and TIDs following intrathecal treatment.

Outcome	Number of patients and studies	Results weighed together	Reliability	Comment
**Intrathecal treatment with PID** compared with **intrathecal treatment with TID**				
**Difference VAS/NRS**	*n*=305,<br>3 non-randomised comparative studies (NRSI)	Little or no difference in analgesic effect between external and internal pumps	⊕⊕⊕◯<br>Moderate reliability	The reliability assessment starts at +4 according to the basic rule for NRSI.<br>Risk of bias[a]: –1

[a]**Studies are assessed as having a high and moderate risk of bias, mainly due to the risk of selection and the presence of unknown factors (confounding factors) in the inclusion of patients**
**Conclusion: It is likely that there is little or no difference in pain-relieving effect between external and internal pumps**.

Table J9. GRADE assessment of evidence reliability from three studies(26, 29, 32) on complications and side effects of intrathecal treatment: PIDs versus TIDs.

**Outcome**	**Number of patients and studies**	**Results weighed together**	**Reliability**	**Comment**
**Intrathecal treatment with PID** compared with **intrathecal treatment with TID**				
**Difference regarding complications and side effects**	*n*=305, 3 non-randomised comparative studies (NRSI)	There is little or no difference in the risk of complications and side effects between PIDs and TIDs.	⊕⊕◯◯ Low reliability	The reliability assessment starts at +4 according to the basic rule for NRSI. Risk of bias[a]: –1 Precision[b]: –1
[a]**See table J8.** [b]**Varying levels of detail and incomplete descriptions of complications both before and after intrathecal treatment.**				

Conclusion: It is possible that there is little or no difference in the risk of complications and side effects between external and internal pumps.

Appendix K: Figures

Figure K1. Effect of intrathecal treatment with an external pump for cancer pain on dosage of systemic opioids (numbers in parentheses shows study population).

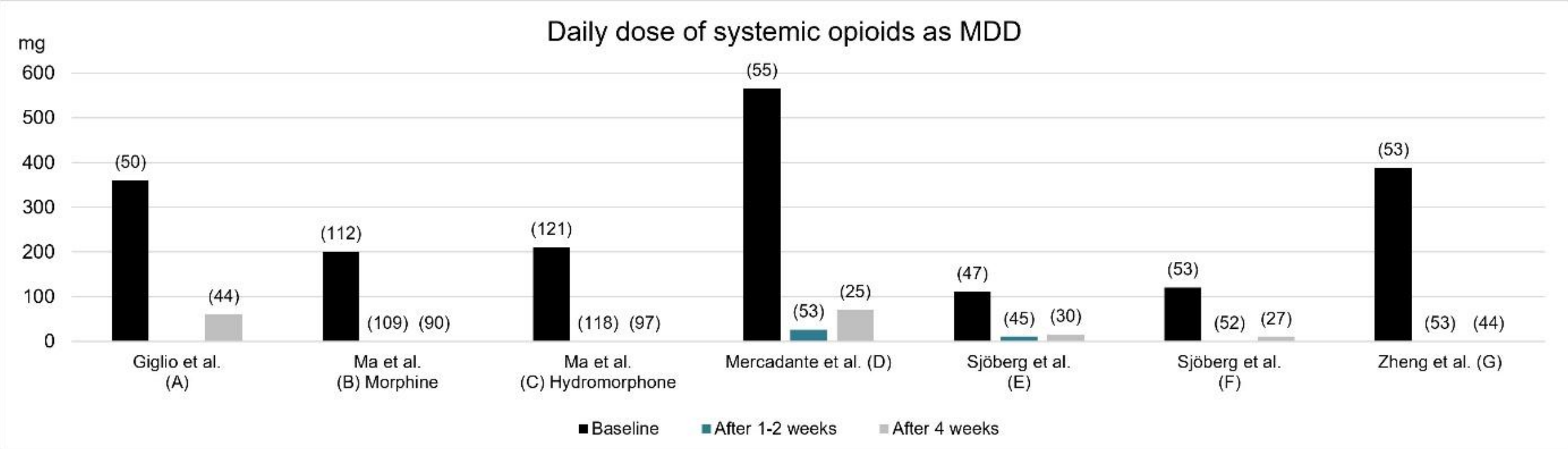

OME: Oral morphine equivalent daily dose. (A) Giglio et al.(14); (B&C) Ma et al.(11); (D) Mercadante et al.(22); (E) Sjöberg et al.(23); (F) Sjöberg et al.(24); (G) Zheng et al.(13). For clarity, only central estimates are displayed in the figures; measures of variability and uncertainty reported in the original studies are presented in Appendix H.

Figure K2. Effect of intrathecal treatment with external pump on health-related QoL for three studies(10, 13, 14). Numbers in parentheses refer to the study population at the measurement point.

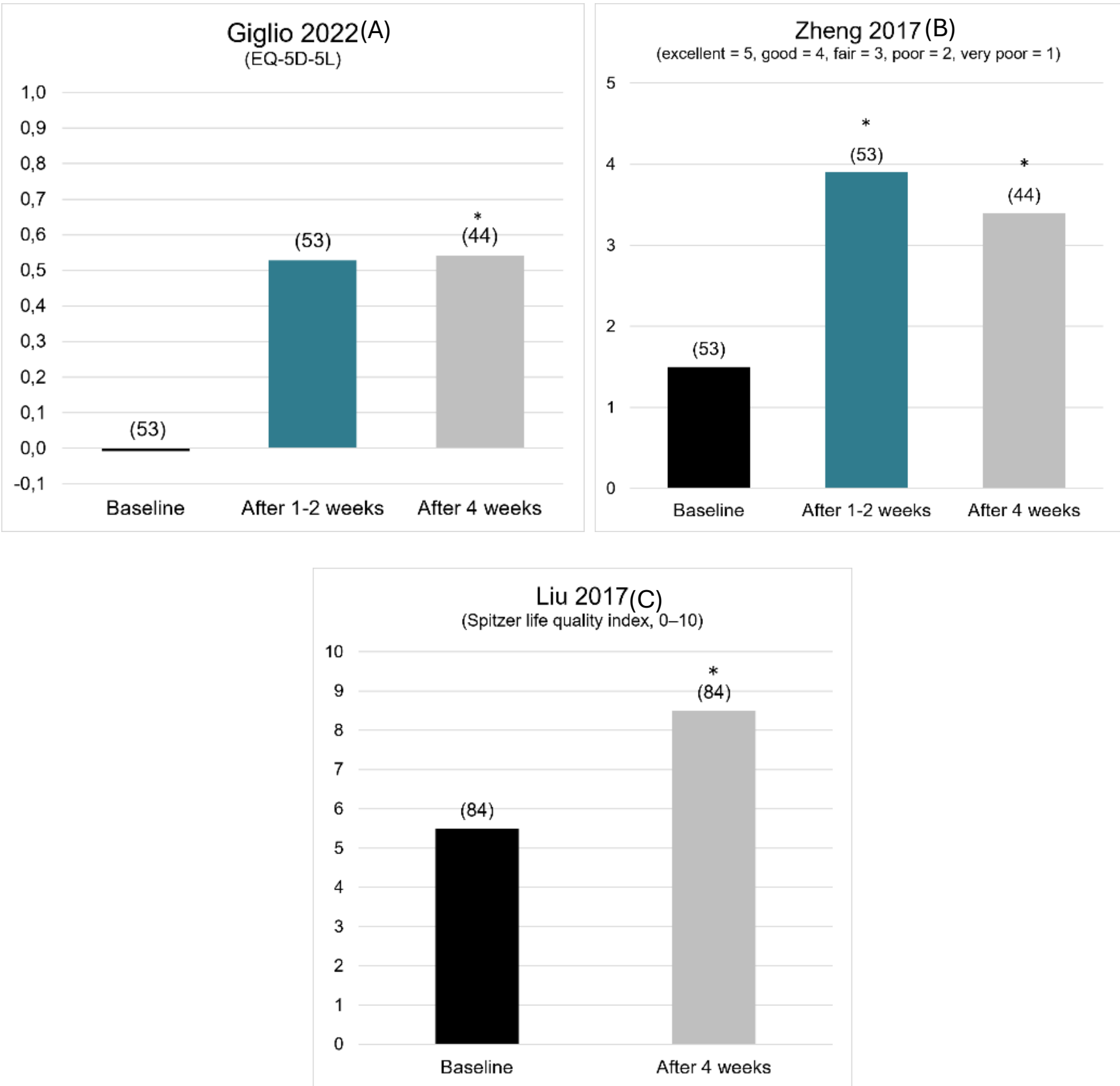

**Significant* p < *0.05. (A) Giglio et al.(14); (B) Zheng et al.(13); (C) Liu et al.(10). For clarity, only central estimates are displayed in the figures; measures of variability and uncertainty reported in the original studies are presented in Appendix H.*

Figure K3. Effect on depression and anxiety during intrathecal treatment with an external pump

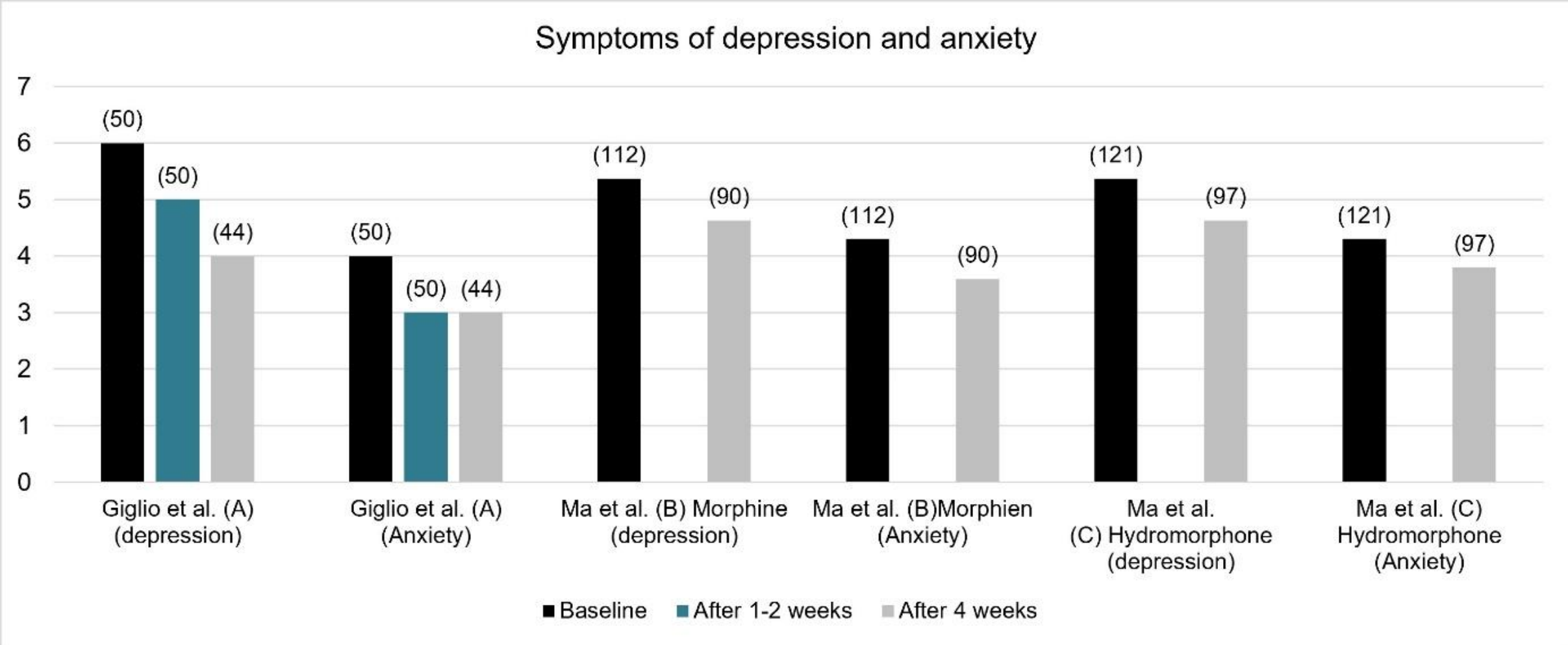

*. *Statistically significant difference at significance level 0.05. When * is missing, a significance calculation has not been performed. (A) Giglio et al.(14) (B&C) Ma et al.(11). For clarity, only central estimates are displayed in the figures; measures of variability and uncertainty reported in the original studies are presented in Appendix H.*

Figure K4. Effect on functional capacity during intrathecal treatment with an external pump; the changes are not significant. For this outcome measure, there is no measurement point after 4 weeks.

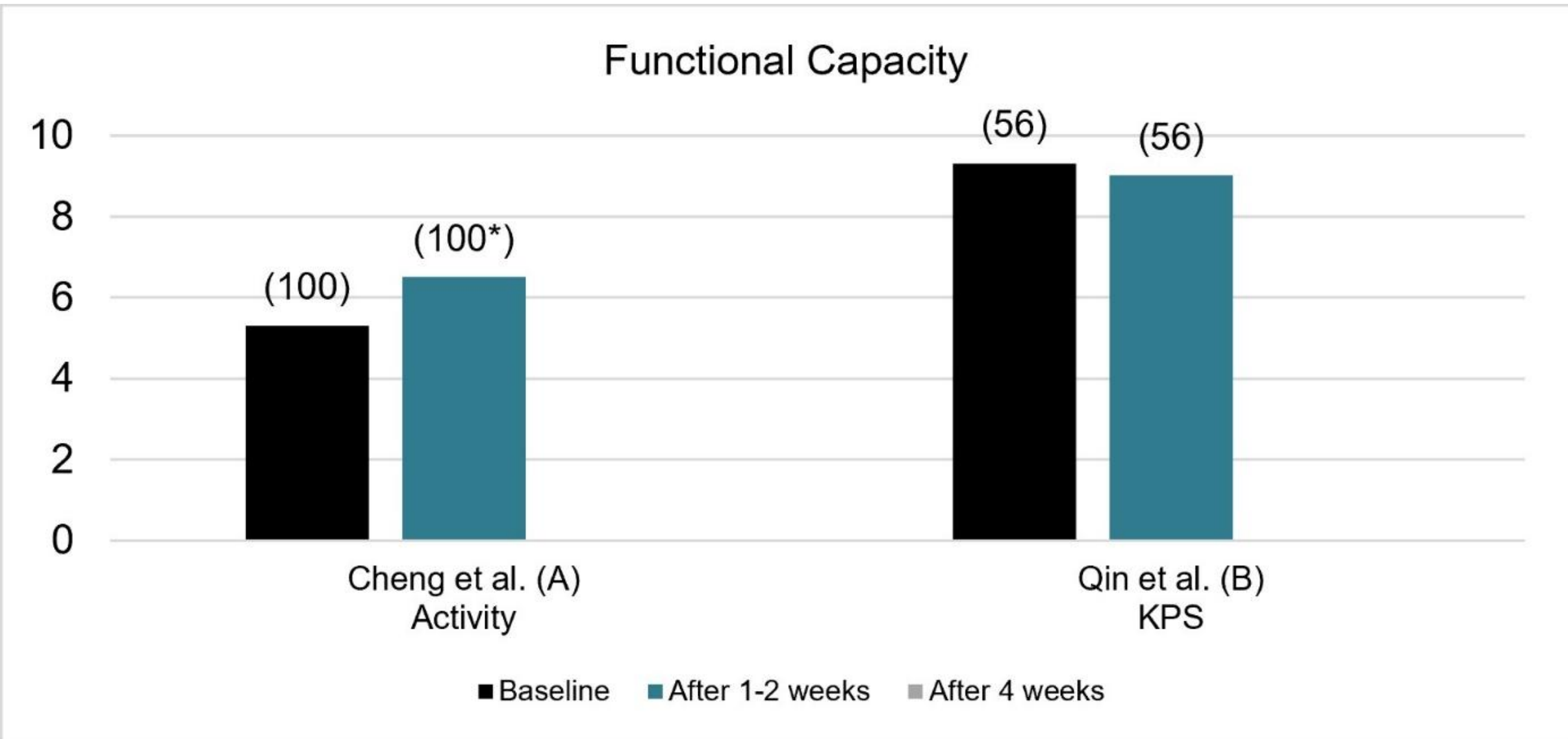

*KPS: Karnofski Performance Scale; *Number missing, assumed to be the same as at baseline measurement. (A) Cheng et al.(25); (B) Qin et al.(26). Functional capacity was assessed using different instruments (a study-specific activity scale and the Karnofsky Performance Scale [KPS]); details are provided in Appendix H.*

For clarity, only central estimates are displayed in the figures; measures of variability and uncertainty reported in the original studies are presented in Appendix H.

Appendix L: PRISMA check lists

L-A: Abstract check list

Section and Topic	Item #	Checklist item	Reported (Yes/No)
**TITLE**			
Title	1	Identify the report as a systematic review.	Yes
**BACKGROUND**			
Objectives	2	Provide an explicit statement of the main objective(s) or question(s) the review addresses.	Yes
**METHODS**			
Eligibility criteria	3	Specify the inclusion and exclusion criteria for the review.	Yes
Information sources	4	Specify the information sources (e.g. databases, registers) used to identify studies and the date when each was last searched.	Yes
Risk of bias	5	Specify the methods used to assess risk of bias in the included studies.	No
Synthesis of results	6	Specify the methods used to present and synthesise results.	Yes
**RESULTS**			
Included studies	7	Give the total number of included studies and participants and summarise relevant characteristics of studies.	Yes
Synthesis of results	8	Present results for main outcomes, preferably indicating the number of included studies and participants for each. If meta-analysis was done, report the summary estimate and confidence/credible interval. If comparing groups, indicate the direction of the effect (i.e. which group is favoured).	Yes
**DISCUSSION**			
Limitations of evidence	9	Provide a brief summary of the limitations of the evidence included in the review (e.g. study risk of bias, inconsistency and imprecision).	Yes
Interpretation	10	Provide a general interpretation of the results and important implications.	Yes

Section and Topic	Item #	Checklist item	Reported (Yes/No)
**OTHER**			
Funding	11	Specify the primary source of funding for the review.	
Registration	12	Provide the register name and registration number.	No

L-B: Main check list

Section and Topic	Item #	Checklist item	Location where item is reported
**TITLE**			
Title	1	Identify the report as a systematic review.	P1
**ABSTRACT**			
Abstract	2	See the PRISMA 2020 for Abstracts checklist.	P1-2
**INTRODUCTION**			
Rationale	3	Describe the rationale for the review in the context of existing knowledge.	P5
Objectives	4	Provide an explicit statement of the objective(s) or question(s) the review addresses.	P4-5
**METHODS**			
Eligibility criteria	5	Specify the inclusion and exclusion criteria for the review and how studies were grouped for the syntheses.	P6-8
Information sources	6	Specify all databases, registers, websites, organisations, reference lists and other sources searched or consulted to identify studies. Specify the date when each source was last searched or consulted.	P5-6<br>P32-36
Search strategy	7	Present the full search strategies for all databases, registers and websites, including any filters and limits used.	P5-6& appendix A&B
Selection process	8	Specify the methods used to decide whether a study met the inclusion criteria of the review, including how many reviewers screened each record and each report retrieved, whether they worked independently, and if applicable, details of automation tools used in the process.	P5-6, Appendix A-F
Data collection process	9	Specify the methods used to collect data from reports, including how many reviewers collected data from each report, whether they worked independently, any processes for obtaining or confirming data from study investigators, and if applicable, details of automation tools used in the process.	P6-8 & Appendix A-F
Data items	10a	List and define all outcomes for which data were sought. Specify whether all results that were compatible with each outcome domain in each study were sought (e.g. for all measures, time points, analyses), and if not, the methods used to decide which results to collect.	P 9-10
	10b	List and define all other variables for which data were sought (e.g. participant and intervention characteristics, funding sources). Describe any assumptions made about any missing or unclear information.	P 7, 20
Study risk of bias assessment	11	Specify the methods used to assess risk of bias in the included studies, including details of the tool(s) used, how many reviewers assessed each	P 9, Appendix C, D1

Section and Topic	Item #	Checklist item	Location where item is reported
		study and whether they worked independently, and if applicable, details of automation tools used in the process.	
Effect measures	12	Specify for each outcome the effect measure(s) (e.g. risk ratio, mean difference) used in the synthesis or presentation of results.	P 9-10
Synthesis methods	13a	Describe the processes used to decide which studies were eligible for each synthesis (e.g. tabulating the study intervention characteristics and comparing against the planned groups for each synthesis (item #5)).	Appendix E-F
	13b	Describe any methods required to prepare the data for presentation or synthesis, such as handling of missing summary statistics, or data conversions.	P 5-6, appaendix A
	13c	Describe any methods used to tabulate or visually display results of individual studies and syntheses.	Available on demand
	13d	Describe any methods used to synthesize results and provide a rationale for the choice(s). If meta-analysis was performed, describe the model(s), method(s) to identify the presence and extent of statistical heterogeneity, and software package(s) used.	P 5-6, 9
	13e	Describe any methods used to explore possible causes of heterogeneity among study results (e.g. subgroup analysis, meta-regression).	P 9-10
	13f	Describe any sensitivity analyses conducted to assess robustness of the synthesized results.	P 21, appendix I-J
Reporting bias assessment	14	Describe any methods used to assess risk of bias due to missing results in a synthesis (arising from reporting biases).	Appendix C
Certainty assessment	15	Describe any methods used to assess certainty (or confidence) in the body of evidence for an outcome.	P2, 9, 21, appendix J, tables 4-9
**RESULTS**			
Study selection	16a	Describe the results of the search and selection process, from the number of records identified in the search to the number of studies included in the review, ideally using a flow diagram.	P 11-19
	16b	Cite studies that might appear to meet the inclusion criteria, but which were excluded, and explain why they were excluded.	Apendix D-G
Study characteristics	17	Cite each included study and present its characteristics.	P 11-19
Risk of bias in studies	18	Present assessments of risk of bias for each included study.	Appendix D
Results of individual studies	19	For all outcomes, present, for each study: (a) summary statistics for each group (where appropriate) and (b) an effect estimate and its precision (e.g. confidence/credible interval), ideally using structured tables or plots.	Appendix H, K
Results of syntheses	20a	For each synthesis, briefly summarise the characteristics and risk of bias among contributing studies.	P 9, appendix C
	20b	Present results of all statistical syntheses conducted. If meta-analysis was done, present for each the summary estimate and its precision (e.g. confidence/credible interval) and measures of statistical heterogeneity. If comparing groups, describe the direction of the effect.	P 21-26
	20c	Present results of all investigations of possible causes of heterogeneity among study results.	P 27, 30
	20d	Present results of all sensitivity analyses conducted to assess the robustness of the synthesized results.	P 21, appendix I

Section and Topic	Item #	Checklist item	Location where item is reported
Reporting biases	21	Present assessments of risk of bias due to missing results (arising from reporting biases) for each synthesis assessed.	P 30
Certainty of evidence	22	Present assessments of certainty (or confidence) in the body of evidence for each outcome assessed.	P20-24, appendix J, tables 4-9
**DISCUSSION**			
Discussion	23a	Provide a general interpretation of the results in the context of other evidence.	P 29-30
	23b	Discuss any limitations of the evidence included in the review.	P 29-30
	23c	Discuss any limitations of the review processes used.	P 29-30
	23d	Discuss implications of the results for practice, policy, and future research.	P 29
**OTHER INFORMATION**			
Registration and protocol	24a	Provide registration information for the review, including register name and registration number, or state that the review was not registered.	Review was not registered
	24b	Indicate where the review protocol can be accessed, or state that a protocol was not prepared.	Protocol was not prepared
	24c	Describe and explain any amendments to information provided at registration or in the protocol.	
Support	25	Describe sources of financial or non-financial support for the review, and the role of the funders or sponsors in the review.	Ingrid & Sverker Persson foundation
Competing interests	26	Declare any competing interests of review authors.	
Availability of data, code and other materials	27	Report which of the following are publicly available and where they can be found: template data collection forms; data extracted from included studies; data used for all analyses; analytic code; any other materials used in the review.	Data and code available on request
